# Investigation on Hydrazonobenzenesulfonamides as Human Carbonic Anhydrase I, II, IX and XII Inhibitors

**DOI:** 10.3390/molecules28010091

**Published:** 2022-12-22

**Authors:** Davide Moi, Serena Vittorio, Andrea Angeli, Gianfranco Balboni, Claudiu T. Supuran, Valentina Onnis

**Affiliations:** 1 Department of Life and Environmental Sciences, University of Cagliari, University Campus, S.P. 8 CA, 09042 Monserrato, Italy; 2 Dipartimento di Scienze Farmaceutiche, Università degli Studi di Milano,Via Mangiagalli 25, 20133 Milano, Italy; 3 Laboratorio di Chimica Bioinorganica, Polo Scientifico Neurofarba Department, Università Degli Studi di Firenze, Room 188, Via della Lastruccia 3, Sesto Fiorentino, 50019 Florence, Italy

**Keywords:** sulfonamides, hydrazones, carbonic anhydrase enzyme inhibition

## Abstract

A small series of hydrazonobenzenesulfonamides was designed, synthesized and studied for their human carbonic anhydrase (hCA) inhibitory activity. The synthesized compounds were evaluated against hCA I, II, IX and XII isoforms using acetazolamide (AAZ) as the standard inhibitor. Various hydrazonosulfonamide derivatives showed inhibitory activity at low nanomolar levels with selectivity against the cytosolic hCA II isoform, as well as the transmembrane, tumor-associated enzymes hCA IX and XII. The most potent and selective hydrazones **8**, **9**, **10**, **11**, **19** and **24** were docked into isoforms I, II, IX and XII to better understand their activity and selectivity for the different CA isoforms.

## 1. Introduction

Human carbonic anhydrases (hCAs) belong to the alpha CA class and are zinc-containing enzymes which catalyze the reversible hydration of carbon dioxide to bicarbonate and a proton (CO_2_ + H_2_O ⇆ HCO_3_^−^ + H^+^). To date, 15 diverse isoforms have been identified, which differ in molecular structure, catalytic properties, organ and tissue distribution as well as subcellular localization, and ability to interact with different inhibitor classes [1]. Different physiological and pathological functions are regulated by hCAs, such as electrolyte secretion and pH regulation, gluconeogenesis, osteoclast functionality and tumorigenicity [1,2]. Thus, hCA inhibitors can be used for the treatment of glaucoma, obesity, neuropathic pain, arthritis, Alzheimer’s disease and cancer. The cytosolic isoforms hCA I and hCA II are widely expressed in erythrocytes, eyes, gastrointestinal tract, osteoclasts and kidney cells [2]. Some hCA II inhibitors are currently used as anti-glaucoma drugs and as mild diuretics [3]. However, the hCA I and II isoforms are considered off-targets in the development of inhibitors for the transmembrane, tumor-associated hCA IX and XII isoforms [4]. Despite the prominent role of hCA II in the regulation of intraocular pressure, anti-glaucoma agents also decrease bicarbonate and aqueous humor secretion by inhibition of hCA I, IV and XII [5,6,7]. hCA II activity has been also associated with neurodegeneration and aging processes [8] and altered hCA II expression constitutes a biomarker of carcinogenesis in some human tumors [9]. The transmembrane hCA IX and XII, among others, are involved in tumorigenesis (hCA IX and XII) and glaucoma (hCA II and XII). Under physiological conditions, the expression of hCA isoform IX is restricted to the basolateral membrane of epithelial cells of the gastric, intestinal and gallbladder mucosa [10] as well as in specialized cells of the ovaries and testes, in some sites of the peripheral and central nervous systems and in mesodermal cells of the placenta and cartilaginous tissues of joints [11,12]. A very low expression of hCA XII is present in tissues such as kidney, pancreas, colon, prostate, ovary, testis, endometrium, lung, brain and colon [13,14,15,16] and is associated with rapid tumor growth, metastasis and infiltration into surrounding normal tissues [17,18,19]. 

Hydrazones have been extensively studied due to their wide biological activities [20,21,22,23,24,25,26,27] while a urea group is a commonly incorporated tailing linker in benzenesulfonamide CA inhibitors. The hydrazone group is a urea-bioisostere, thus different groups have reported successful approaches to inhibit CA by hydrazone-based compounds [28,29,30,31,32,33,34,35,36]. 

As a continuation of our efforts in the design and synthesis of new potent and selective hCA Is [37,38,39,40,41], this study presents a small library of hydrazonobenzenesulfonamide-based compounds. The target compounds are endowed with a benzenesulfonamide-type zinc-binding group (ZBG) connected by a carbonyl bridge to a piperidine ring, which provides an appropriate orientation and connects the two tails with both the hydrophilic and hydrophobic halves of the active site. The hydrazone moiety can also improve the flexibility and hydrophilicity of the designed compounds to improve hCA inhibitory activity through favorable interactions with the specific residues in the hydrophilic part of the active site.

## 2. Results

### 2.1. Chemistry

**Scheme 1:**
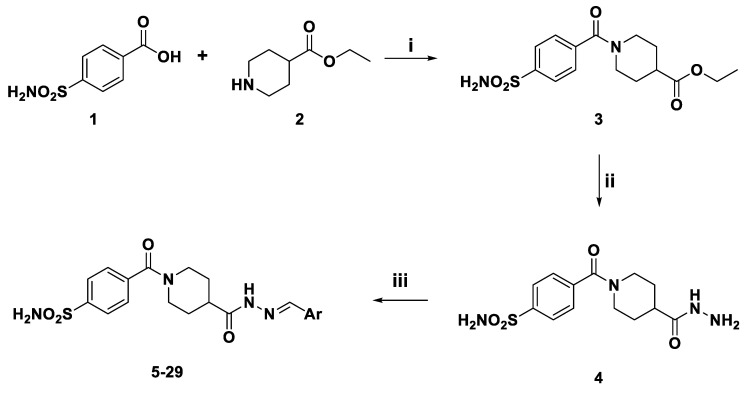
General synthetic procedure for hydrazonosulfonamides **5**–**29**. Reagents and conditions: (i) EDCI, HOBt, dry CH_3_CN r.t. 12h, 77% yield; (ii) NH_2_NH_2_·H_2_O, absolute EtOH, reflux 3h, 78% yield; substituted arylaldehydes, absolute EtOH, reflux 6h, 52–98% yield.

The synthesis of hydrazonobenzenesulfonamide derivatives **5**–**29** reported here was performed as highlighted in Scheme 1, starting from 4-sulfamoylbenzoic acid **1**. By using our previously reported synthetic procedure [42], the coupling of sulfanilamide **1** with ethyl piperidine-4-carboxylate **2** in the presence of 1-(3-dimethylaminopropyl)-3-ethylcarbodiimide hydrochloride (EDCI) in a dry acetonitrile solution (MeCN) produced ethyl 1-(4-sulfamoylbenzoyl)piperidine-4-carboxylate **3**. This ester was treated with hydrazine hydrate in absolute ethanol (EtOH) to obtain the corresponding 4-(4-(hydrazinecarbonyl)piperidine-1-carbonyl)benzenesulfonamide **4**. Treatment of the hydrazide **4** with an equimolecular amount of appropriately substituted aldehydes in EtOH solution furnished the hydrazones **5**–**29** with yields of 52–98%. The spectral data are consistent with the assigned structure. 

According to the literature, the presence of a downfield resonating (10.89–11.73 ppm) CH=N signal can exclusively account for the formation of E isomers [43]. The signal is split due to hindered amide rotation around the C–N bond of the acyl fragment that generates two conformers [43].

### 2.2. Carbonic Anhydrase Inhibition

Sulfonamidohydrazones **5**–**29** were tested for their ability to inhibit four human (h) CA isoforms involved in crucial physiologic/pathologic processes: the cytosolic hCA I and II and the transmembrane, tumor-associated hCA IX and XII. Table 1 shows the inhibition data of the derivatives reported here and the sulfonamide acetazolamide (AAZ) as the standard inhibitor tested using a stopped-flow CO_2_ hydrase assay [44]. 

The following structure activity relationship (SAR) may be noted regarding the inhibition data in Table 1.

The slow cytosolic isoform hCA I was effectively inhibited by a few of the new hydrazonosulfonamides reported here. The strongest inhibitory effects were observed for compounds **5**, **7**, **12**, **13**, **27**, and **29** which had inhibition constants ranging between 18.5 and 45.5 nM (better than acetazolamide, AAZ, which has a K_I_ of 250 nM against this isoform). These hydrazones possess the following benzylidene moieties: phenyl, 2-methoxyphenyl, 2,5-dimethoxyphenyl, 2,4,5-trimetoxyphenyl, 2,6-dichlorobenzyl and 4-(dimethylamino)phenyl. Another group of derivatives, including **8**, **9**, **11**, **14**, **16**, **19**, **20**, and **22**–**26**, showed medium-potency inhibitory activity against hCA I, with KIs in the range of 334–800 nM (Table 1). They possess 3-methoxyphenyl, 4-methoxyphenyl, 3,4-dimethoxyphenyl, 3,4,5-trimethoxyphenyl, 2-methoxynaphthyl, 3-fluorophenyl, 4-fluorophenyl, 4-nitrophenyl, chorophenyl and dichlorophenyl benzylidene moieties.

The dominant cytosolic isoform hCA II was also effectively inhibited by many of the hydrazones reported here. The highly effective inhibitors were **5**-**7**, **9**, **12**, **13**, **22**, **23** and **25**-**29**, with KIs in the range of 1.75–6.65 nM. They possess phenyl, 4-methylphenyl, 4-methoxyphenyl, 2,5-dimethoxyphenyl, 2,4,5-trimethoxyphenyl, 4-nitrophenyl, 2-chlorophenyl, 2,4-dichlorophenyl, 2,5-dichlorophenyl, 2,6-dichlorophenyl and 4-(dialkylamino)phenyl benzylidene moieties. As for hCA I, small structural changes between hydrazones led to dramatic differences in their inhibitory activity. For example, an impressive difference of inhibitory power was observed for the two isomeric nitrophenyl-substituted compounds **21** and **22**, with the 4-nitrophenyl derivative being a 9-fold better inhibitor than its isomer with the 3-nitrophenyl group. Medium potency inhibitors included the following hydrazones: **8**, **11**, **14**, **15**, **17**–**19**, **21**, and **24** (KIs in the range of 25.2–92.4 nM) whereas the weakest hCA II inhibitor in the series was **10** (KI 159.6 nM).

hCA IX was generally effectively inhibited by the new hydrazones reported here. The best inhibitory effects were observed using compounds **10**, **11**, **17**, **19** and **23**, which had inhibition constants ranging between 15.4 and 23.4 nM (better than the reference inhibitor AAZ, which has a KI of 25 nM against this isoform). These hydrazones contain the following benzylidene moieties: 2,4-dimethoxyphenyl, 3,4-dimethoxyphenyl, 2-methoxynaphthyl, 3-fluorophenyl, 2-chlorophenyl 2,4,5-trimetoxyphenyl, 2,6-dichlorobenzyl and 4-(dimethylamino)phenyl benzylidene moieties. Another group of derivatives, including **5**–**8**, **12**-**15**, **18**, **20**, **22**, **24**–**27** and **29** showed high-potency inhibitory activity against hCA IX, with KIs in the range of 34.3–67.5 nM. Only hydrazones **9**, **16**, **21** and **28** showed micromolar inhibitory potencies (KIs in the range of 123–320 nM, see the Supplementary Materials). As for hCA I and hCA II, small structural changes between hydrazones led to marked differences in their inhibitory activity. For example, a large difference in inhibitory potency was observed for the two isomeric methoxyphenyl-substituted compounds **8** and **9**, with the 3-methoxyphenyl derivative being an 8-fold better inhibitor than its isomer with the 4-methoxyphenyl group.

A rather similar situation as was noted above for hCA IX has also been observed for the inhibition of the second transmembrane isoform investigated here, hCA XII. Almost all hydrazone derivatives showed efficient inhibition of this isoform (KIs in the range of 8.05–68.7 nM). The most active hydrazones incorporate the following benzylidene moieties: 2-methoxyphenyl, 2,4-dimethoxyphenyl, 3,4-dimethoxyphenyl, naphthyl and 3-fluorophenyl. The shift of the 2-methoxy group in compound **7** to the 4-position to produce hydrazone **9** results in a approx. 12-fold decrease in activity.

The unsubstituted hydrazone **5** showed no significant difference in activity towards the four tested hCA isoforms. The introduction of a 4-methyl group gave the highly hCA II-selective hydrazone **6**. The replacement of the methyl by a 4-methoxy group produced a reduction in hCA II selectivity (compound **9**). The shift of the methoxy group to the 2-position caused a reduction of hCA II selectivity and enhancement of hCA XII selectivity. The comparison of compound **8** and **9** activities indicated that the shift of the methoxy group to the 3-position resulted in an approx. 10- and 5-fold increase in activity against hCA IX and hCA XII, respectively, as well as an approx. 10-fold reduction in hCA II activity. The 2,4-dimethoxybenzilidene (compound **10**) and the 3,4-dimethoxybenzilidene (compound **11**) moieties produced high activity and selectivity against the tumor-associated hCA IX and hCA XII isoforms with hydrazone **10** as the most selective of the series towards hCA XII. The introduction of a 2-bromine atom to compound **11** (hydrazone **15**) produced a slight decrease in activity on all the tested hCA isoforms. The introduction of a second methoxy group to the 5-position on compound **7** to give hydrazone **12** led to a reduction in hCA XII activity and to an improvement in hCA II activity as compared to **7**. Similar inhibition data were shown by hydrazone **13** bearing a third methoxy group at the 4-position. The shift of the methoxy group to the 3-position (hydrazone **14**) increased the selectivity towards hCA IX and hCA XII. The naphthyl derivative **16** showed about a 420-fold hCA XII/hCA I and 22-fold hCA XII/CA II increase in selectivity. The introduction of a methoxy group to produce hydrazone **17** increased hCA IX in activity and reduced hCA XII selectivity as compared with **16**. The chlorine-substituted hydrazones displayed high hCA II selectivity except for the 3-chlorobenzylidene derivative **24** that showed about a 3-fold hCA XII/hCA II and 20-fold hCA XII/hCA I increase in selectivity.

The replacement of the methyl group on compound **6** by a 4-trifluoromethyl group reduced hCA I and hCA II selectivity (compound **18**). The replacement of the 4-trifluoromethyl with a 4-fluorine atom (hydrazone **20**) caused a further reduction in hCA I activity and increase of inhibitory activity against the other three hCA isoforms. The shift of the fluorine into the 3-position (hydrazone **19**) produced a large increase in both activity and selectivity towards hCA IX and hCA XII. The 3-nitro-substituted hydrazone **21** showed a reduction in activity against hCA I, hCA IX and hCA XII as compared to the 3-fluorine-substituted hydrozone, while the high hCA II selectivity was maintained.

### 2.3. Molecular Docking

**Figure 1:**
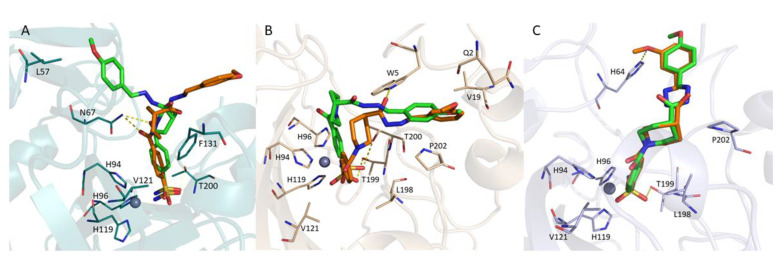
Binding modes of compounds **8** (orange sticks) and **9** (green sticks): (**A**) binding mode of **8** and **9** to hCA II active site; (**B**) binding mode of **8** and **9** to hCA IX active site; (**C**) binding mode of **8** and **9** to hCA XII active site. The residues of the binding site involved in the interactions with the inhibitors are shown as sticks. H-bond interactions are displayed as yellow dashed lines.

Due to their potent activity profile, the binding mode of compounds **8**, **9**, **10**, **11**, **19** and **24** to hCA isoforms II, IX and XII was investigated by docking simulations in order to rationalize the hCA isoform selectivity observed for these derivatives. All the compounds occupied the active sites of the studied hCA isoforms with the NH^-^ and C=O groups of the sulfonamide moiety involved in the coordination of the Zn ion and in H-bonds with Thr199 or 200, respectively. As discussed above, derivative **9** showed the best selectivity profile towards hCA II which decreased when the 4-methoxy group was shifted to the 3-position as in compound **8**, for which an increase in affinity towards hCA IX and hCA XII was observed. Both compounds might occupy the hCA II-binding pocket by engaging hydrophobic interactions with Phe131 and a H-bond with Asn67 (Figure 1A). 

The main differences between the two binding poses rely on the different orientations assumed by the benzensulfonamide moiety and the tail of the molecules. More specifically, in compound **8** the former could be involved in hydrophobic contacts with Val121 while no interactions were observed for the tail portion. In the case of inhibitor **9**, the arylsulfonamide moiety might be stabilized by π-stacking interactions with His94, while the tail is oriented towards Leu57 which establishes hydrophobic contacts with the methoxy group and might contribute to further stabilization of the binding to the hCA II active site. When bound to hCA IX (Figure 1B), hydrazone **9** might form hydrophobic contacts with Pro199 through the phenyl ring of the tail, while additional interactions were observed for the 3-methoxy-substituted analogue **8** which might elicit several hydrophobic contacts with Leu198, Val121, Pro202, Gln2 and Val19 and a H-bond with Trp5, thus explaining its higher affinity towards hCA IX compared to compound **9**. Despite these two inhibitors showing similar binding modes to the hCA XII active site (Figure 1C), the 3-methoxy group of hydrazone **8** allowed the formation of a convenient H-bond with His64 that was not observed for hydrazone **9** and this might be the reason for the higher inhibitory activity of derivative **8** towards hCA XII.

**Figure 2:**
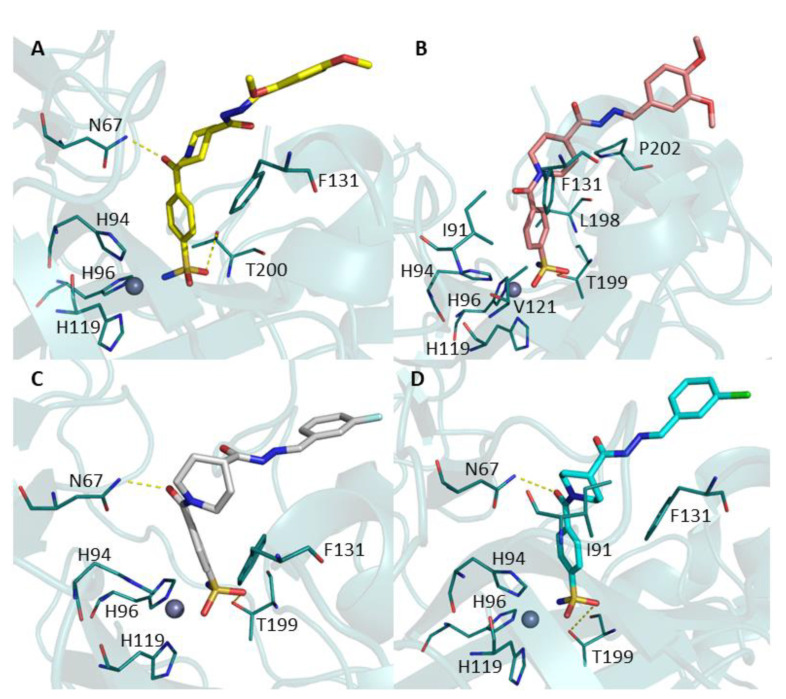
Docking poses of hydrazones **10**, **11**, **19** and **24** to hCA II active site: (**A**) hydrazone **10**; (**B**) hydrazone **11**; (**C**) hydrazone **19**; (**D**) hydrazone **24**. The residues of the binding site involved in the interactions with the inhibitors are shown as sticks. H-bond interactions are displayed as yellow dashed lines.

Hydrazones **10**, **11**, **19** and **24** displayed a good selectivity towards the tumor-associated isoforms hCA IX and hCA XII over hCA II. As shown in Figure 2, these hydrazones might bind the hCA II active site by establishing hydrophobic interactions with Phe131 through the piperidine ring, which might also be stabilized by additional contacts with Pro202 and Ile91 in the case of compounds **11** and **24**, respectively. 

**Figure 3:**
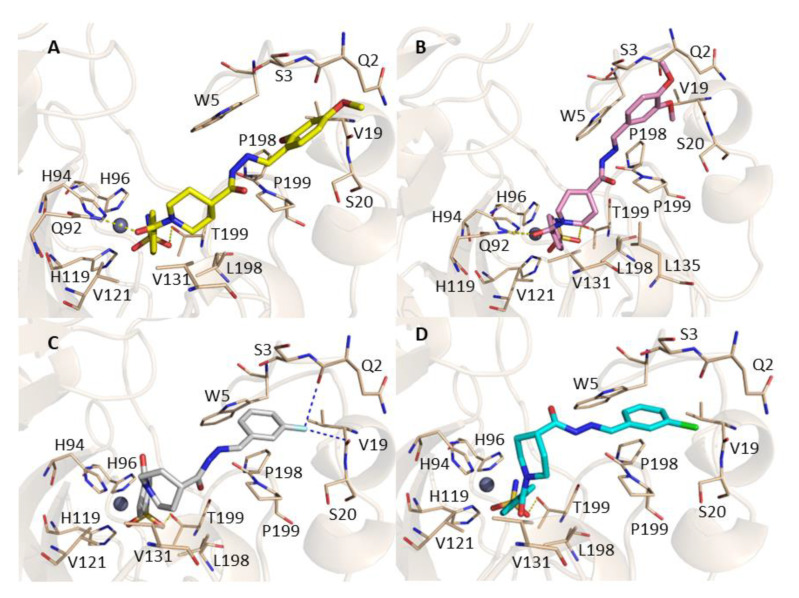
Docking poses of hydrazones **10**, **11**, **19**, and **24** to hCA IX active site: (**A**) hydrazone **10**; (**B**) hydrazone **11**; (**C**) hydrazone **19**; (**D**) hydrazone **24**. The residues of the binding site involved in the interactions with the inhibitors are shown as sticks. H-bond and halogen bond interactions are displayed as yellow and blue dashed lines, respectively.

Other hydrophobic interactions were observed between the benzensulfonamide moiety of hydrazone **11** and Leu198 and Val121. Furthermore, hydrazones **10**, **19** and **24** might engage a H-bond between the side chain of Asn67 and the amide carbonyl group. No relevant interactions were observed between the tails of derivatives **10**, **11**, **19** and **24** and the rim of the hCA II active site. Conversely, a major stabilization of these molecules’ tails was observed in the docking simulations performed with the hCA IX and hCA XII binding pockets, which can explain the major affinity of these compounds towards the tumor-associated isoforms. Specifically, the four inhibitors might assume similar binding orientations within the hCA IX active site (Figure 3), with the benzensulfonamide moiety and the piperidine ring engaging hydrophobic contacts with Val121, Leu198 and Val131, while the tails occupy a region lined by Gln2, Ser3, Trp5, Val19, Ser20, Pro198 and Pro199, which are involved in hydrophobic interactions with these inhibitors. Moreover, hydrazones **10** and **11** could form a H-bond with Gln92 through the amide carbonyl group (Figure 3A,B), while the fluorine atom of compound **19** might elicit halogen bonds with Val19 and Gln2 (Figure 3C). These additional interactions exerted by hydrazones **10**, **11** and **19** might explain their high potency towards hCA IX compared to derivative **24**.

**Figure 4:**
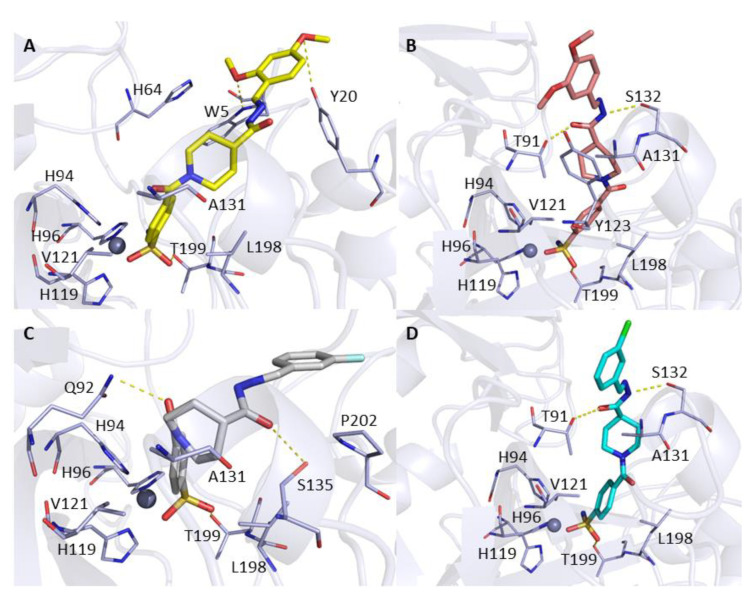
Docking poses of hydrazones **10**, **11**, **19** and **24** to hCA XII active site: (**A**) hydrazone **10**; (**B**) hydrazone **11**; (**C**) hydrazone **19**; (**D**) hydrazone **24**. The residues of the binding site involved in the interactions with the inhibitors are shown as sticks. H-bonds are displayed as yellow dashed lines.

Regarding hCA XII (Figure 4), compounds **10**, **11**, **19** and **24** could establish hydrophobic contacts with Leu198 and Val121 through their phenyl moiety and with Ala131 through the piperidine ring. Moreover, derivatives **11** and **24** might elicit H-bonds with Thr91 and Ser132 through one of their carbonyl group and hydrazone nitrogen (Figure 4B,D), while compound **10** might engage H-bonds with Trp5 and Tyr20 through its two methoxy groups (Figure 4A). Conversely, hydrazone **19** interacts with Gln92 and Ser135 by forming hydrogen bonds with its two carbonyl groups (Figure 4C). Finally, hydrophobic interactions were detected between: (i) His64 and the 2-methoxy group of compound **10**; (ii) Tyr123 and the 3-methoxy group of hydrazone **11;** and (iii) Pro202 and the 3-fluorophenyl ring of compound **19**.

## 3. Materials and Methods

### 3.1. Chemistry

All commercially available solvents and reagents were used without further purification. ^1^H NMR spectra were recorded using an Inova 500 spectrometer (Varian, Palo Alto, CA, USA). ^13^C NMR spectra were recorded using a Bruker Advance III HD 600 (Bruker, Bremen, Germany) at 126 MHz. The chemical shifts (δ) are reported in parts per million downfield from tetramethylsilane (TMS), which was used as an internal standard. The spectra were recorded in hexadeuteriodimethylsulphoxide (DMSO-d_6_). Infrared spectra were recorded using a Vector 22 spectrometer (Bruker, Bremen, Germany) in Nujol mulls. The main bands are given in cm^−1^. Positive-ion electrospray ionization (ESI) mass spectra were recorded using a double-focusing MAT 95 instrument (Finnigan, Waltham, MA, USA) with BE geometry. Melting points (mp) were determined with a SMP1 Melting Point apparatus (Stuart Scientific, Stone, UK) and are uncorrected. All products reported showed ^1^H NMR spectra in agreement with the assigned structures. The purity of the tested compounds was determined by combustion elemental analyses conducted by the Microanalytical Laboratory of the Chemistry Department of the University of Ferrara with a MT-5 CHN recorder elemental analyzer (Yanagimoto, Kyoto, Japan) and the values found were within 0.4% of theoretical values. 4-(4-(Hydrazinecarbonyl)piperidine-1-carbonyl)benzenesulfonamide (**4**) was synthesized as previously reported [42].

#### 3.1.1. General Procedure for the Preparation of (E)-4-(4-(2-benzylidenehydrazinecarbonyl)piperidine-1-carbonyl)benzenesulfonamides (5-25)

A mixture of 4-(4-(hydrazinecarbonyl)piperidine-1-carbonyl)benzenesulfonamide (**4**) (0.33 g, 1 mmol) and the appropriate arylaldehyde (1 mmol) in absolute ethanol (EtOH) (5 mL) was refluxed overnight. After cooling, the formed precipitate was filtered off and recrystallized from EtOH to give the hydrazones **5**–**29**.

##### (E)-4-(4-(2-benzylidenehydrazinecarbonyl)piperidine-1-carbonyl)benzenesulfonamide (5) 

Yield 97%, M.p.> 250°C. ^1^H NMR (DMSO-d_6_) 1.59 (br s, 2H, CH), 1.73 (br s, 1H, CH), 1.88 (br s, 1H, CH), 2.99 (br s, 1H, CH), 3.20 (br s, 1H, CH), 3.44 (br s, 1H, CH), 3.50 (br s, 1H, CH), 4.48 (br s, 1H, CH), 7.39 (m, 3H, Ar), 7.42 (s, 2H, NH_2_), 7.57 (d, J = 8.0 Hz, 2H, Ar), 7.65 (m, 2H, Ar), 7.87 (d, J = 8.0 Hz, 2H, Ar), 7.97, 8.18 (s, 1H, NH), 11.23, 11.37 (s, 1H, CH). IR (Nujol) 3209, 3066, 1676, 1561 cm^−1^. Elemental analysis: calculated for C_20_H_22_N_4_O_4_S (414.48) %C 57.96, %H 5.35, %N 13.52, found %C 58.01, %H 5.33, %N 13.48. m/z 415.

##### (E)-4-(4-(2-(4-methylbenzylidene)hydrazinecarbonyl)piperidine-1-carbonyl)benzenesulfonamide (6) 

Yield 89%, M.p. >250°C. ^1^H NMR (DMSO-*d_6_*) 1.58 (br s, 2H, CH), 1.71 (br s, 1H, CH), 1.87 (br s, 1H, CH), 2.31 (s, 3H, CH_3_), 2.91 (br s, 1H, CH), 3.17 (br s, 1H, CH), 3.42 (br s, 1H, CH), 3.52 (br s, 1H, CH), 4.48 (br s, 1H, CH), 7.23 (d, *J* = 8.5 Hz, 2H, Ar), 7.42 (s, 2H, NH_2_), 7.53 (d, *J* = 8.0 Hz, 2H, Ar), 7.57 (d, *J* = 8.5 Hz, 2H, Ar), 7.87 (d, *J* = 8.0 Hz, 2H, Ar), 7.95, 8.14 (s, 1H, NH), 11.15, 11.30 (s, 1H, CH). IR (Nujol) 3328, 3227, 1675, 1557 cm^−1^. Elemental analysis: calculated for C_21_H_24_N_4_O_4_S (428.50) %C 58.86, %H 5.65, %N 13.07, found %C 58.80, %H 5.67, %N 13.11. m/z 429.

##### (E)-4-(4-(2-(2-methoxybenzylidene)hydrazinecarbonyl)piperidine-1-carbonyl)benzenesulfonamide (7) 

Yield 77%, M.p. 234-235°C. ^1^H NMR (DMSO-d_6_) 1.58 (br s, 2H, CH), 1.71 (br s, 1H, CH), 1.86 (br s, 1H, CH), 2.87 (br s, 1H, CH), 3.20 (br s, 1H, CH), 3.42 (br s, 1H, CH), 3.51 (br s, 1H, CH), 3.82 (s, 3H, OCH_3_), 4.47 (br s, 1H, CH), 6.97 (t, J = 7.0 Hz, 1H, Ar), 7.07 (d, J = 8.0 Hz, 1H, Ar), 7.37 (d, J = 7.0 Hz, 1H, Ar), 7.42 (s, 2H, NH_2_), 7.57 (d, J = 8.0 Hz, 2H, Ar), 7.77 (t, J = 7.5 Hz, 1H, Ar), 7.86 (d, J = 7.5 Hz, 2H, Ar), 8.32, 8.52 (s, 1H, NH), 11.19, 11.36 (s, 1H, CH). IR (Nujol) 3333, 3287, 3234, 1643, 1528 cm^−1^. Elemental analysis: calculated for C_21_H_24_N_4_O_5_S (444.50) %C 56.74, %H 5.44, %N 12.60, found %C 56.79, %H 5.43, %N 12.57. m/z 445.

##### (E)-4-(4-(2-(3-methoxybenzylidene)hydrazinecarbonyl)piperidine-1-carbonyl)benzenesulfonamide (8) 

Yield 95%, M.p. 243-244°C. ^1^H NMR (DMSO-*d_6_*) 1.59 (br s, 2H, CH), 1.72 (br s, 1H, CH), 1.87 (br s, 1H, CH), 2.88 (br s, 1H, CH), 3.25 (br s, 1H, CH), 3.43 (br s, 1H, CH), 3.53 (br s, 1H, CH), 3.77 (s, 3H, OCH_3_), 4.47 (br s, 1H, CH), 6.97 (s, 1H, Ar), 7.20 (d, *J* = 7.5 Hz, 2H, Ar), 7.32 (m, 1H, Ar), 7.42 (s, 2H, NH_2_), 7.57 (d, *J* = 8.0 Hz, 2H, Ar), 7.87 (d, *J* = 7.0 Hz, 2H, Ar), 7.94, 8.15 (s, 1H, NH), 11.25, 11.38 (s, 1H, CH). ^13^C NMR (DMSO-*d_6_*) 28.3, 28.9, 40.4, 46.9, 55.4, 104.6, 107.7, 111.1, 126.3, 127.7, 129.9, 139.9, 141.3, 145.1, 155.7, 160.1, 168.3, 174.2. IR (Nujol) 3210, 3067, 1678, 1560 cm^−1^. Elemental analysis: calculated for C_21_H_24_N_4_O_5_S (444.50) %C 56.74, %H 5.44, %N 12.60, found %C 56.69, %H 5.45, %N 12.65. m/z 445.

##### (E)-4-(4-(2-(4-methoxybenzylidene)hydrazinecarbonyl)piperidine-1-carbonyl)benzenesulfonamide (9) 

Yield 81%, M.p. 228-230°C. ^1^H NMR (DMSO-*d_6_*) 1.58 (br s, 2H, CH), 1.71 (br s, 1H, CH), 1.87 (br s, 1H, CH), 2.87 (br s, 1H, CH), 3.21 (br s, 1H, CH), 3.42 (br s, 1H, CH), 3.52 (br s, 1H, CH), 3.78 (s, 3H, OCH_3_), 4.48 (br s, 1H, CH), 6.97 (d, *J* = 7.0 Hz, 2H, Ar), 7.43 (s, 2H, NH_2_), 7.59 (m, 4H, Ar), 7.88 (d, *J* = 8.0 Hz, 2H, Ar), 7.93, 8.12 (s, 1H, NH), 11.11, 11.25 (s, 1H, CH). ^13^C NMR (DMSO-*d_6_*) 28.4, 37.9, 41.1, 55.8, 141.8, 126.4, 127.33, 127.3, 127.8, 128.8, 129.0. 140.0, 143.3, 145.1, 146.7, 161.0, 161.2, 168.3, 170.42, 175.6. IR (Nujol) 3310, 3248, 1667, 1556 cm^−1^. Elemental analysis: calculated for C_21_H_24_N_4_O_5_S (444.50) %C 56.74, %H 5.44, %N 12.60, found %C 56.79, %H 5.46, %N 12.56. m/z 445.

##### (E)-4-(4-(2-(2,4-dimethoxybenzylidene)hydrazinecarbonyl)piperidine-1-carbonyl)benzenesulfonamide (10) 

Yield 98%, M.p. 208-210°C. ^1^H NMR (DMSO-*d_6_*) 1.57 (br s, 2H, CH), 1.70 (br s, 1H, CH), 1.85 (br s, 1H, CH), 2.92 (br s, 1H, CH), 3.08 (br s, 1H, CH), 3.39 (br s, 1H, CH), 3.51 (br s, 1H, CH), 3.81 (s, 6H, OCH_3_), 4.46 (br s, 1H, CH), 6.58 (d, *J* = 8.5 Hz, 2H, Ar), 7.42 (s, 2H, NH_2_), 7.56 (d, *J* = 8.0 Hz, 2H, Ar), 7.70 (s, 1H, Ar), 7.87 (d, *J* = 8.0 Hz, 2H, Ar), 8.22, 8.42 (s, 1H, NH), 11.04, 11.22 (s, 1H, CH). IR (Nujol) 3348, 3251, 1659, 1558 cm^−1^. Elemental analysis: calculated for C_22_H_26_N_4_O_6_S (474.16) %C 55.68, %H 5.52, %N 11.81, found %C 55.74, %H 5.51, %N 11.78. m/z 475.

##### (E)-4-(4-(2-(3,4-dimethoxybenzylidene)hydrazinecarbonyl)piperidine-1-carbonyl)benzenesulfonamide (11)

Yield 76%, M.p. >250°C. ^1^H NMR (DMSO-*d_6_*) 1.55 (br s, 2H, CH), 1.72 (br s, 1H, CH), 1.83 (br s, 1H, CH), 2.92 (br s, 1H, CH), 3.11 (br s, 1H, CH), 3.36 (br s, 1H, CH), 3.54 (br s, 1H, CH), 3.82 (s, 6H, OCH_3_), 4.45 (br s, 1H, CH), 6.56 (d, *J* = 8.5 Hz, 2H, Ar), 7.44 (s, 2H, NH_2_), 7.51 (d, *J* = 8.0 Hz, 2H, Ar), 7.72 (s, 1H, Ar), 7.88 (d, *J* = 8.0 Hz, 2H, Ar), 8.23, 8.41 (s, 1H, NH), 11.06, 11.19 (s, 1H, CH). IR (Nujol) 3285, 3246, 1663, 1559 cm^−1^. Elemental analysis: calculated for C_22_H_26_N_4_O_6_S (474.53) %C 55.68, %H 5.52, %N 11.81, found %C 55.62, %H 5.53, %N 11.85. m/z 475.

##### (E)-4-(4-(2-(2,5-dimethoxybenzylidene)hydrazinecarbonyl)piperidine-1-carbonyl)benzenesulfonamide (12) 

Yield 97%, M.p. 210–211°C. ^1^H NMR (DMSO-*d_6_*) 1.57 (br s, 2H, CH), 1.72 (br s, 1H, CH), 1.87 (br s, 1H, CH), 2.87 (br s, 1H, CH), 3.19 (br s, 1H, CH), 3.44 (br s, 1H, CH), 3.51 (br s, 1H, CH), 3.72 (s, 3H, OCH_3_), 3.78 (s, 3H, OCH_3_), 4.47 (br s, 1H, CH), 6.96 (d, *J* = 8.5 Hz, 1H, Ar), 7.03 (d, *J* = 8.0 Hz, 1H, Ar), 7.27 (m, 1H, Ar), 7.41 (s, 2H, NH_2_), 7.56 (d, *J* = 8.5 Hz, 2H, Ar), 7.86 (d, *J* = 8.5 Hz, 2H, Ar), 8.27, 8.49 (s, 1H, NH), 11.21, 11.38 (s, 1H, CH). IR (Nujol) 3328, 3257, 3073, 1673 cm^−1^. Elemental analysis: calculated for C_22_H_26_N_4_O_6_S (474.53) %C 55.68, %H 5.52, %N 11.81, found %C 55.63, %H 5.50, %N 11.77. m/z 475.

##### (E)-4-(4-(2-(2,4,5-trimethoxybenzylidene)hydrazinecarbonyl)piperidine-1-carbonyl)benzenesulfonamide (13) 

Yield 72%, M.p. >250°C. ^1^H NMR (DMSO-*d_6_*) δ 1.57 (br s, 2H, CH), 1.71 (br s, 1H, CH), 1.83 (br s, 1H, CH), 2.87 (br s, 1H, CH), 3.18 (br s, 1H, CH), 3.40 (br s, 1H, CH), 3.52 (br s, 1H, CH), 3.72 (s, 3H, OCH_3_), 3.81 (s, 6H, OCH_3_), 4.47 (br s, 1H, CH), 6.70 (s, 1H, Ar), 7.26 (s, 1H, Ar), 7.42 (s, 2H, NH_2_), 7.57 (d, *J* = 8.0 Hz, 2H, Ar), 7.87 (d, *J* = 8.0 Hz, 2H, Ar), 8.22, 8.44 (s, 1H, NH), 11.06, 11.22 (s, 1H, CH). IR (Nujol) 3322, 3223, 1695 cm^−1^. Elemental analysis: calculated for C_23_H_28_N_4_O_7_S (504.56) %C 54.75, %H 5.59, %N 11.10, found %C 54.69, %H 5.60, %N 11.14. m/z 505.

##### (E)-4-(4-(2-(3,4,5-trimethoxybenzylidene)hydrazinecarbonyl)piperidine-1-carbonyl)benzenesulfonamide (14)

Yield 76%, M.p. >250°C. ^1^H NMR (DMSO-*d_6_*) δ 1.59 (br s, 2H, CH), 1.73 (br s, 1H, CH), 1.86 (br s, 1H, CH), 2.52 (br s, 1H, CH), 3.10 (br s, 1H, CH), 3.41 (br s, 1H, CH), 3.52 (br s, 1H, CH), 3.67 (s, 3H, OCH_3_), 3.68 (s, 6H, OCH_3_), 4.49 (br s, 1H, CH), 6.96 (s, 2H, Ar), 7.42 (s, 2H, NH_2_), 7.57 (d, *J* = 8.0 Hz, 2H, Ar), 7.87 (d, *J* = 8.5 Hz, 2H, Ar), 7.89, 8.10 (s, 1H, NH), 11.27, 11.35 (s, 1H, CH). IR (Nujol) 3354, 3252, 1680, 1550 cm^−1^. Elemental analysis: calculated for C_23_H_28_N_4_O_7_S (504.56) %C 54.75, %H 5.59, %N 11.10, found %C 54.81, %H 5.58, %N 11.08. m/z 505.

##### (E)-4-(4-(2-(2-bromo-3,4-dimethoxybenzylidene)hydrazinecarbonyl)piperidine-1-carbonyl)benzenesulfonamide (15)

Yield 94%, M.p.> 250°C. ^1^H NMR (DMSO-*d_6_*) 1.58 (br s, 2H, CH), 1.72 (br s, 1H, CH), 1.88 (br s, 1H, CH), 2.89 (br s, 1H, CH), 3.21 (br s, 1H, CH), 3.42 (br s, 1H, CH), 3.52 (br s, 1H, CH), 3.75 (s, 3H, OCH_3_), 3.86 (s, 3H, OCH_3_), 4.47 (br s, 1H, CH), 7.33 (d, *J* = 6.5 Hz, 1H, Ar), 7.42 (s, 2H, NH_2_), 7.44 (m, 1H, Ar), 7.57 (d, *J* = 8.0 Hz, 2H, Ar), 7.87 (d, *J* = 6.5 Hz, 2H, Ar), 7.89, 8.09 (s, 1H, NH), 11.32, 11.46 (s, 1H, CH). IR (Nujol) 3358, 3246, 3060, 1680, 1548 cm^−1^. Elemental analysis: calculated for C_22_H_25_BrN_4_O_6_S (553.43) %C 47.75, %H 4.55, %N 10.12, found %C 47.79, %H 4.54, %N 10.15. m/z 555.

##### (E)-4-(4-(2-((naphthalen-2-yl)methylene)hydrazinecarbonyl)piperidine-1-carbonyl)benzenesulfonamide (16)

Yield 71%, M.p. 220-222°C. ^1^H NMR (DMSO-*d_6_*) δ 1.64 (br s, 2H, CH), 1.79 (br s, 1H, CH), 1.96 (br s, 1H, CH), 2.92 (br s, 1H, CH), 3.24 (br s, 1H, CH), 3.43 (br s, 1H, CH), 3.56 (br s, 1H, CH), 4.52 (br s, 1H, CH), 7.44 (s, 2H, NH_2_), 7.58 (m, 6H, Ar), 7.86 (m, 3H, Ar), 7.98 (d, *J* = 8.2 Hz, 2H, Ar), 8.81, 8.99 (s, 1H, NH), 11.30, 11.48 (s, 1H, CH). IR (Nujol) 3253, 3069, 1680, 1619 cm^−1^. Elemental analysis: calculated for C_24_H_24_N_4_O_5_S (464.54) %C 62.05, %H 5.21, %N 12.06, found %C 60.76, %H 5.31, %N 11.29. m/z 464.

##### (E)-4-(4-(2-((1-methoxynaphthalen-2-yl)methylene)hydrazinecarbonyl)piperidine-1-carbonyl)benzenesulfonamide (17)

Yield 67%, M.p. >250°C. ^1^H NMR (DMSO-*d_6_*) δ 1.64 (br s, 2H, CH), 1.79 (br s, 1H, CH), 1.90 (br s, 1H, CH), 2.96 (br s, 1H, CH), 3.20 (br s, 1H, CH), 3.41 (br s, 1H, CH), 3.56 (br s, 1H, CH), 3.96 (s, 3H, OCH_3_), 4.52 (br s, 1H, CH), 7.42 (s, 2H, NH_2_), 7.48 (d, *J* = 9.0 Hz, 2H, Ar), 7.54 (m, 3H, Ar), 7.58 (d, *J* = 8.5 Hz, 2H, Ar), 7.90 (m, 2H, Ar), 8.01 (m, 1H, Ar), 8.72, 8.87 (s, 1H, NH), 11.26, 11.45 (s, 1H, CH). IR (Nujol) 3351, 3329, 1658 cm^−1^. Elemental analysis: calculated for C_25_H_26_N_4_O_5_S (494.56) %C 60.71, %H 5.30, %N 11.33, found %C 60.76, %H 5.31, %N 11.29. m/z 495.

##### (E)-4-(4-(2-(4-(trifluoromethyl)benzylidene)hydrazinecarbonyl)piperidine-1-carbonyl)benzenesulfonamide (18)

Yield 96%, M.p. >250°C. ^1^H NMR (DMSO-d_6_) 1.60 (br s, 2H, CH), 1.73 (br s, 1H, CH),1.88 (br s, 1H, CH), 2.92 (br s, 1H, CH), 3.22 (br s, 1H, CH), 3.48 (br s, 1H, CH), 3.52 (br s, 1H, CH), 4.48 (br s, 1H, CH), 7.43 (s, 2H, NH_2_), 7.57 (d, J = 8.0 Hz, 2H, Ar), 7.76 (d, J = 7.5 Hz, 2H, Ar), 7.87 (m, 2H, Ar), 7.88 (m, 2H, Ar), 8.06, 8.21 (s, 1H, NH), 11.43, 11.58 (s, 1H, CH). IR (Nujol) 3364, 3240, 1680, 1556 cm^−1^. Elemental analysis: calculated for C_21_H_21_F_3_N_4_O_4_S (482.48) %C 52.28, %H 4.39, %N 11.61, found %C 52.33, %H 4.40, %N 11.58. m/z 483.

##### (E)-4-(4-(2-(3-fluorobenzylidene)hydrazinecarbonyl)piperidine-1-carbonyl)benzenesulfonamide (19) 

Yield 79%, M.p. 214-215 °C. ^1^H NMR (DMSO-d_6_) 1.58 (br s, 2H, CH), 1.72 (br s, 1H, CH), 1.89 (br s, 1H, CH), 2.88 (br s, 1H, CH), 3.23 (br s, 1H, CH), 3.44 (br s, 1H, CH), 3.51 (br s, 1H, CH), 4.47 (br s, 1H, CH), 7.22 (s, 1H, Ar), 7.43 (s, 2H, NH_2_), 7.48 (m, 3H, Ar), 7.57 (d, J = 8.0 Hz, 2H, Ar), 7.87 (d, J = 7.0 Hz, 2H, Ar), 7.99, 8.18 (s, 1H, NH), 11.33, 11.49 (s, 1H, CH). IR (Nujol) 3284, 3209, 1680, 1565 cm^−1^. Elemental analysis: calculated for C_20_H_21_FN_4_O_4_S (432.47) %C 55.54, %H 4.89, %N 12.96, found %C 55.49, %H 4.90, %N 12.99. m/z 433.

##### (E)-4-(4-(2-(4-fluorobenzylidene)hydrazinecarbonyl)piperidine-1-carbonyl)benzenesulfonamide (20) 

Yield 84%, M.p.> 250°C. ^1^H NMR (DMSO-d_6_) 1.58 (br s, 2H, CH), 1.71 (br s, 1H, CH), 1.86 (br s, 1H, CH), 2.88 (br s, 1H, CH), 3.21 (br s, 1H, CH), 3.43 (br s, 1H, CH), 3.52 (br s, 1H, CH), 4.48 (br s, 1H, CH), 7.25 (d, *J* = 8.5 Hz, 2H, Ar), 7.43 (s, 2H, NH_2_), 7.57 (d, *J* = 8.5 Hz, 2H, Ar), 7.71 (d, *J* = 7.0 Hz, 2H, Ar), 7.86 (d, *J* = 8.0 Hz, 2H, Ar); 7.98, 8.18 (s, 1H, NH); 11.23, 11.38 (s, 1H, CH). IR (Nujol) 3350, 3252, 1667, 1624 cm^−1^. Elemental analysis: calculated for C_20_H_21_FN_4_O_4_S (432.47) %C 55.54, %H 4.89, %N 12.96, found %C 55.49, %H 4.87, %N 13.00. m/z 433.

##### (E)-4-(4-(2-(3-nitrobenzylidene)hydrazinecarbonyl)piperidine-1-carbonyl)benzenesulfonamide (21) 

Yield 87%, M.p. >250°C. ^1^H NMR (DMSO-d_6_) 1.59 (br s, 2H, CH), 1.73 (br s, 1H, CH), 1.89 (br s, 1H, CH), 2.87 (br s, 1H, CH), 3.25 (br s, 1H, CH), 3.44 (br s, 1H, CH), 3.47 (br s, 1H, CH), 4.48 (br s, 1H, CH), 7.42 (s, 2H, NH_2_), 7.57 (d, J = 8.0 Hz, 2H, Ar), 7.71 (s, 1H, Ar), 7.87 (d, J = 8.0 Hz, 2H, Ar), 8.01 (d, J = 7.5 Hz, 2H, Ar), 8.30 (m, 1H, Ar), 8.43, 8.49 (s, 1H, NH), 11.47, 11.64 (s, 1H, CH). IR (Nujol) 3328, 3213, 3069, 1680, 1564 cm^−1^. Elemental analysis: calculated for C_20_H_21_N_5_O_6_S (459.48) %C 52.28, %H 4.61, %N 15.24, found %C 52.33, %H 4.59, %N 15.20. m/z 453.

##### (E)-4-(4-(2-(4-nitrobenzylidene)hydrazinecarbonyl)piperidine-1-carbonyl)benzenesulfonamide (22) 

Yield 87%, M.p.> 250°C. ^1^H NMR (DMSO-*d_6_*) 1.60 (br s, 2H, CH), 1.73 (br s, 1H, CH), 1.89 (br s, 1H, CH), 2.89 (br s, 1H, CH), 3.22 (br s, 1H, CH), 3.47 (br s, 1H, CH), 3.53 (br s, 1H, CH), 4.48 (br s, 1H, CH), 7.43 (s, 2H, NH_2_), 7.57 (d, *J* = 8.5 Hz, 2H, Ar), 7.87 (d, *J* = 8.0 Hz, 2H, Ar), 7.92 (m, 2H, Ar), 8.26 (d, *J* = 8.5 Hz, 2H, Ar), 8.08, 8.28 (s, 1H, NH), 11.54, 11.69 (s, 1H, CH). IR (Nujol) 3287, 3085, 1668, 1588 cm^−1^. Elemental analysis: calculated for C_20_H_21_N_5_O_6_S (459.48) %C 52.28, %H 4.61, %N 15.24, found %C 52.35, %H 4.60, %N 15.21. m/z 460.

##### (E)-4-(4-(2-(2-chlorobenzylidene)hydrazinecarbonyl)piperidine-1-carbonyl)benzenesulfonamide (23) 

Yield 81%, M.p. >250°C. ^1^H NMR (DMSO-*d_6_*) 1.59 (br s, 2H, CH), 1.73 (br s, 1H, CH), 1.88 (br s, 1H, CH), 2.99 (br s, 1H, CH), 3.20 (br s, 1H, CH), 3.44 (br s, 1H, CH), 3.50 (br s, 1H, CH), 4.48 (br s, 1H, CH), 7.39 (m, 2H, Ar), 7.42 (s, 2H, NH_2_), 7.49 (t, *J* = 7.5 Hz, 1H, Ar), 7.57 (d, *J* = 8.5 Hz, 2H, Ar), 7.87 (d, *J* = 7.5 Hz, 2H, Ar), 7.87 (m, 1H, Ar), 8.37, 8.58 (s, 1H, NH), 11.43, 11.62 (s, 1H, CH). IR (Nujol) 3240, 3074, 1677, 1538 cm^−1^. Elemental analysis: calculated for C_20_H_21_ClN_4_O_4_S (448.92) %C 53.51, %H 4.72, %N 12.48, found %C 53.57, %H 4.71, %N 12.44. m/z 449.

##### (E)-4-(4-(2-(3-chlorobenzylidene)hydrazinecarbonyl)piperidine-1-carbonyl)benzenesulfonamide (24) 

Yield 94%, M.p.> 250°C. ^1^H NMR (DMSO-*d_6_*) 1.58 (br s, 2H, CH), 1.72 (br s, 1H, CH), 1.87 (br s, 1H, CH), 2.91 (br s, 1H, CH), 3.22 (br s, 1H, CH), 3.44 (br s, 1H, CH), 3.52 (br s, 1H, CH), 4.47 (br s, 1H, CH), 7.42 (s, 2H, NH_2_), 7.44 (m, 2H, Ar), 7.57 (d, *J* = 8.0 Hz, 2H, Ar), 7.61 (m, 1H, Ar), 7.70 (m, 1H, Ar), 7.87 (d, *J* = 8.0 Hz, 2H, Ar), 7.97, 8.16 (s, 1H, NH), 11.34, 11.51 (s, 1H, CH). IR (Nujol) 3298, 3218, 3070, 1680, 1562 cm^−1^. Elemental analysis: calculated for C_20_H_21_ClN_4_O_4_S (448.92) %C 53.51, %H 4.72, %N 12.48, found %C 53.56, %H 4.73, %N 12.44. m/z 449.

##### (E)-4-(4-(2-(2,4-dichlorobenzylidene)hydrazinecarbonyl)piperidine-1-carbonyl)benzenesulfonamide (25) 

Yield 98%, M.p. 234-235°C. ^1^H NMR (DMSO-*d_6_*) 1.58 (br s, 2H, CH), 1.72 (br s, 1H, CH), 1.87 (br s, 1H, CH), 2.89 (br s, 1H, CH), 3.19 (br s, 1H, CH), 3.43 (br s, 1H, CH), 3.52 (br s, 1H, CH), 4.47 (br s, 1H, CH), 7.42 (s, 2H, NH_2_), 7.47 (m, 1H, Ar), 7.57 (d, *J* = 7.0 Hz, 2H, Ar), 7.68 (m, 1H, Ar), 7.87 (d, *J* = 6.5 Hz, 2H, Ar), 7.92 (m, 1H, Ar), 8.32, 8.53 (s, 1H, NH), 11.47, 11.66 (s, 1H, CH). IR (Nujol) 3253, 3068, 1680, 1552 cm^−1^. Elemental analysis: calculated for C_20_H_20_Cl_2_N_4_O_4_S (483.37) %C 49.70, %H 4.17, %N 11.59, found %C 49.76, %H 4.19, %N 11.54. m/z 483.

##### (E)-4-(4-(2-(2,5-dichlorobenzylidene)hydrazinecarbonyl)piperidine-1-carbonyl)benzenesulfonamide (26) 

Yield 92%, M.p.> 250°C. ^1^H NMR (DMSO-*d_6_*) 1.59 (br s, 2H, CH), 1.72 (br s, 1H, CH), 1.82 (br s, 1H, CH), 2.89 (br s, 1H, CH), 3.23 (br s, 1H, CH), 3.47 (br s, 1H, CH), 3.52 (br s, 1H, CH), 4.47 (br s, 1H, CH), 7.42 (s, 2H, NH_2_), 7.47 (m, 1H, Ar), 7.53 (m, 2H, Ar), 7.57 (d, *J* = 7.5 Hz, 2H, Ar), 7.87 (d, *J* = 7.0 Hz, 2H, Ar), 8.32, 8.52 (s, 1H, NH), 11.51, 11.73 (s, 1H, CH). IR (Nujol) 3304, 3247, 1679, 1573 cm^−1^. Elemental analysis: calculated for C_20_H_20_Cl_2_N_4_O_4_S (483.37) %C 49.70, %H 4.17, %N 11.59, found %C 49.76, %H 4.16, %N 11.55. m/z 483.

##### (E)-4-(4-(2-(2,6-dichlorobenzylidene)hydrazinecarbonyl)piperidine-1-carbonyl)benzenesulfonamide (27) 

Yield 82%, M.p.> 250°C. ^1^H NMR (DMSO-*d_6_*) 1.57 (br s, 2H, CH), 1.73 (br s, 1H, CH), 1.88 (br s, 1H, CH), 2.87 (br s, 1H, CH), 3.11 (br s, 1H, CH), 3.35 (br s, 1H, CH), 3.52 (br s, 1H, CH), 4.48 (br s, 1H, CH), 7.43 (s, 2H, NH_2_), 7.53 (m, 1H, Ar), 7.59 (d, *J* = 7.0 Hz, 2H, Ar), 7.71 (m, 1H, Ar), 7.86 (d, *J* = 6.5 Hz, 2H, Ar), 7.88 (m, 1H, Ar), 8.25, 8.39 (s, 1H, NH), 11.51, 11.67 (s, 1H, CH). IR (Nujol) 3315, 3068, 1668, 1556 cm^−1^. Elemental analysis: calculated for C_20_H_20_Cl_2_N_4_O_4_S (483.37) %C 49.70, %H 4.17, %N 11.59, found %C 49.64, %H 4.19, %N 11.63. m/z 483.

##### (E)-4-(4-(2-(4-(dimethylamino)benzylidene)hydrazinecarbonyl)piperidine-1-carbonyl)benzenesulfonamide (28)

Yield 92%, M.p.> 250°C. ^1^H NMR (DMSO-*d_6_*) 1.58 (br s, 2H, CH), 1.70 (br s, 1H, CH), 1.86 (br s, 1H, CH), 2.86 (br s, 1H, CH), 2.94 (s, 6H, CH_3_), 3.19 (br s, 1H, CH), 3.39 (br s, 1H, CH), 3.52 (br s, 1H, CH), 4.48 (br s, 1H, CH), 6.71 (d, *J* = 8.5 Hz, 2H, Ar), 7.42 (s, 2H, NH_2_), 7.44 (d, *J* = 8.0, 2H, Ar), 7.57 (d, *J* = 8.5 Hz, 2H, Ar), 7.85 (d, *J* = 8.0 Hz, 2H, Ar), 7.88, 8.02 (s, 1H, NH), 10.93, 11.05 (s, 1H, CH). IR (Nujol) 3341, 3242, 1635 cm^−1^. Elemental analysis: calculated for C_22_H_27_N_5_O_4_S (457.55) %C 57.55, %H 5.95, %N 15.31, found %C 57.59, %H 5.96, %N 15.34. m/z 458.

##### (E)-4-(4-(2-(4-(diethylamino)benzylidene)hydrazinecarbonyl)piperidine-1-carbonyl)benzenesulfonamide (29) 

Yield 91%, M.p.> 250°C. ^1^H NMR (DMSO-*d_6_*) 1.08 (t, *J* = 6.5 Hz, 6H, CH_3_), 1.58 (br s, 2H, CH), 1.70 (br s, 1H, CH), 1.85 (br s, 1H, CH), 2.86 (br s, 1H, CH), 3.19 (br s, 1H, CH), 3.39 (br s, 1H, CH), 3.47 (q, *J* = 6.5 Hz, 4H, CH_2_), 3.52 (br s, 1H, CH), 4.48 (br s, 1H, CH), 6.67 (d, *J* = 8.5 Hz, 2H, Ar), 7.42 (m, 4H, NH_2_ and Ar), 7.57 (d, *J* = 8.0 Hz, 2H, Ar), 7.87 (d, *J* = 8.0 Hz, 2H, Ar), 7.82, 7.99 (s, 1H, NH), 10.89, 11.03 (s, 1H, CH). IR (Nujol) 3327, 3218, 1659, 1614 cm^−1^. Elemental analysis: calculated for C_24_H_31_N_5_O_4_S (485.60) %C 59.36, %H 6.43, %N 14.42, found %C 59.42, %H 6.44, %N 14.37. m/z 486.

### 3.2. Carbonic Anhydrase Inhibition

An Applied Photophysics stopped-flow instrument was used for assaying the CA-catalyzed CO_2_ hydration activity [44]. Phenol red (at a concentration of 0.2 mM) was used as an indicator, working at the absorbance maximum of 557 nm, with 20 mM HEPES (pH 7.5) as the buffer, and 20 mM Na_2_SO_4_ (for maintaining a constant ionic strength), following the initial rates of the CA-catalyzed CO_2_ hydration reaction for a period of 10–100 s. The CO_2_ concentrations ranged from 1.7 to 17 mM for the determination of the kinetic parameters and inhibition constants. For each inhibitor at least six traces of the initial 5–10% of the reaction were used for determining the initial velocity. The uncatalyzed rates were determined in the same manner and subtracted from the total observed rates. Stock solutions of inhibitor (0.1 mM) were prepared in 10% DMSO aqueous solution and dilutions up to 0.01 nM were done thereafter with the assay buffer. Inhibitor and enzyme solutions were preincubated together for 15 min at room temperature prior to the assay, in order to allow for the formation of the E-I complex. The inhibition constants were obtained by non-linear least squares methods using PRISM 3 and the Cheng–Prusoff equation, as reported earlier [45,46,47] and represent the mean from at least three different determinations. All CA isoforms were recombinant ones obtained in-house as reported earlier. Their concentrations in the assay system were 5.7–11.9 nM [41,48,49].

### 3.3. Molecular Docking

Molecular docking simulations were carried out by using the crystal structures of CAII (PDB ID 3HS4) [50], CAIX (PDB ID 3IAI) [51] and CAXII (PDB ID 1JD0) [52] in complex with the inhibitor AAZ that was used as a reference compound in this study. The protein structures were prepared as described elsewhere [53]. Ligand structures were submitted to a preliminary conjugate gradient minimization by using AMMP tool implemented in VEGA ZZ software [54]. The minimized structures underwent a conformational search by following a Monte Carlo procedure as implemented in VEGA ZZ. The lowest energy conformation was selected and further optimized by the PM7 semi-empirical method using MOPAC 2016 [55]. The prepared ligands were docked to the CAII, CAIX and CAXII binding pockets by means of GOLD V 5.8.1 [56]. The binding site was defined in order to include all the residues within 10 Å from the co-crystallized ligand. In considering the conserved binding motif of the sulfonamide portion, a scaffold constraint was applied to restrict the docking solutions to those in which this moiety assumes a binding orientation similar to that observed for the native ligand AAZ in the experimental structures. The option “never dock a ligand when a constraint is physically impossible” was checked. All the ligands were submitted to ten genetic algorithms runs following the docking protocols reported elsewhere [57,58] The best docking pose was chosen for the analysis and representation. 

## 4. Conclusions

In the present study, we described a small library of benzenesulfonamides as potential hCA Is, endowed with a benzenesulfonamide-type zinc-binding group (ZBG) connected by a carbonyl bridge to a piperidine ring, which provide an appropriate orientation and connections of the two tails with both the hydrophilic and hydrophobic halves of the active site. The 4-methoxyphenylhydrazone **9** showed the best selectivity against hCA II that might be due the tail orientation producing hydrophobic interactions with the enzyme’s active site. The dimethoxyphenylhydrazone **10** and **11** as well as the 3-halophenylhydrazones **19** and **24** showed the best selectivity against the tumor-associated hCA IX and hCA XII isoforms. The tails of these compounds established hydrophobic interactions with the active site of hCA IX and hCA XII; conversely, no interaction of the tails was possible in the hCA II binding site. Overall, these results indicate that through small structural changes of the benzylidene tail portion of these hCAI inhibitors, it is possible to modulate both their inhibitory activity and hCA isoform selectivity.

## Figures and Tables

**Table 1:** Inhibition data of human CA isoforms I, II, IX and XII by hydrazones **5**–**29** and AAZ as standard inhibitor.

Cmpd	Ar	*K*_I_ (nM) ^1^						
		CAI	CAII	CAIX	CAXII	CAII/CAI Selectivity	CAII/CAIX Selectivity	CAII/CAXII Selectivity
**5**	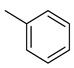	18.5	4.41	34.3	18.0	4.2	7.8	4.1
**6**	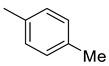	3235	5.62	48.1	13.9	575.6	8.6	2.5
**7**	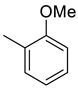	45.5	2.94	55.1	8.55	15.5	18.7	2.9
**8**	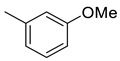	382.5	44.3	38.4	19.4	8.6	0.9	0.4
**9**	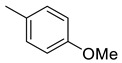	389.5	4.48	320	102	86.9	71,4	22.8
**10**	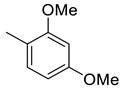	1885	159.6	15.4	9.21	11.8	0.1	0.06
**11**	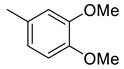	344	55.1	19.6	8.05	6.2	0.4	0.1
**12**	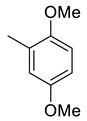	24.3	1.75	44.9	25.8	13.9	25.7	14.7
**13**	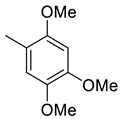	41.8	3.78	52.1	26.1	11.1	13.8	6.9
**14**	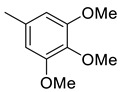	384.5	67.6	39.7	32.4	5.7	0.6	0.5
**15**	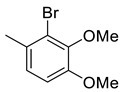	1535	65.5	58.9	36.5	23.4	0.9	0.6
**16**	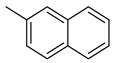	4000	209	123	9.36	19.1	0.6	0.04
**17**	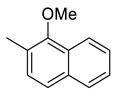	334	25.2	16.4	15.3	13.3	0.6	0.6
**18**	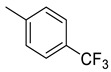	1750	37.4	67.5	55.7	46.8	1.8	1.5
**19**	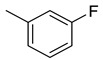	392	92.4	19.7	10.2	4.2	0.2	0.1
**20**	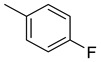	340	17.1	41.6	24.8	19.9	2.4	1.4
**21**	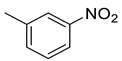	1825	46.2	149	29.4	39.5	3.3	0.6
**22**	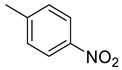	383.5	4.92	64.2	53.1	77.9	13.0	8.2
**23**	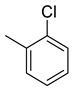	482.5	6.51	23.4	14.4	74.1	3.6	2.2
**24**	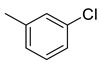	398.5	55.4	36.1	17.3	7.2	0.7	0.3
**25**	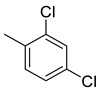	336	6.65	48.7	37.2	50.5	7.3	5.6
**26**	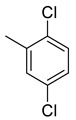	800	3.64	57.7	41.9	219.8	15.8	11.5
**27**	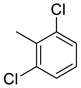	24.6	3.57	54.2	68.7	6.9	15.2	19.2
**28**	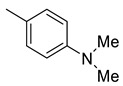	1060	5.39	159	22.9	196.7	29.5	4.2
**29**	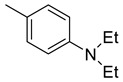	45.0	4.27	64.1	42.1	10.5	15.0	9.9
**AAZ**		250.0	12.0	25.0	5.7	20.8	2.1	0.5

^1^ Mean from three different assays, using a stopped-flow technique (errors were in the range of ±5–10% of the reported values).

## Data Availability

Not applicable.

## Supplementary Materials

The following supporting information can be downloaded at: https://www.mdpi.com/article/10.3390/molecules28010091/s1, ^1^H-NMR spectra of hydrazones **8**, **9**, **10**, **11**, **19** and **24**.

## Sample Availability

Samples of the compounds **5**–**29** are available from the authors.
