# Supplementary material for: Investigation on Hydrazonobenzenesulfonamides as Human Carbonic Anhydrase I, II, IX and XII Inhibitors
Source: Molecules. 2022 Dec 22;28(1):91. doi: 10.3390/molecules28010091 (PMC9822402; doi:10.3390/molecules28010091)

SUPPORTING INFORMATION

Investigation on hydrazonobenzenesulfonamides as human carbonic anhydrase I, II, IX and XII inhibitors

Davide Moi [1], Serena Vittorio[2], Andrea Angeli[3], Gianfranco Balboni[1], Claudiu T. Supuran[3], and Valentina Onnis [1,*]

[1]Department of Life and Environmental Sciences, Unit of Pharmaceutical, Pharmacological and Nutraceutical Sciences, University of Cagliari, Monserrato University Campus, 09042 Monserrato (CA), Italy.

[2] Dipartimento di Scienze Farmaceutiche, Università degli Studi di Milano, Via Mangiagalli 25, Milano, 20133, Italy

[3] Polo Scientifico Neurofarba Department, Laboratorio di Chimica Bioinorganica, Università Degli Studi di Firenze, Room 188, Via della Lastruccia 3, Sesto Fiorentino, Florence, 50019, Italy

* Correspondence to: Prof. Valentina Onnis, Department of Life and Environmental Sciences, Unit of Pharmaceutical, Pharmacological and Nutraceutical Sciences, University of Cagliari, University Campus, 09042 Monserrato (CA), Italy. E-mail vonnis@unica.it

Table of contents

	Pag.
^{1}H NMR Spectra of the hydrazones **5-29.**	**S2-S29**

^{1}H-NMR Spectra of hydrazones 5-29

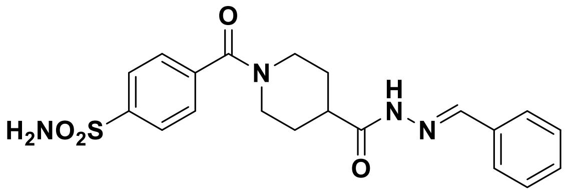

^{1}H NMR, DMSO-*d$_6$*, 5

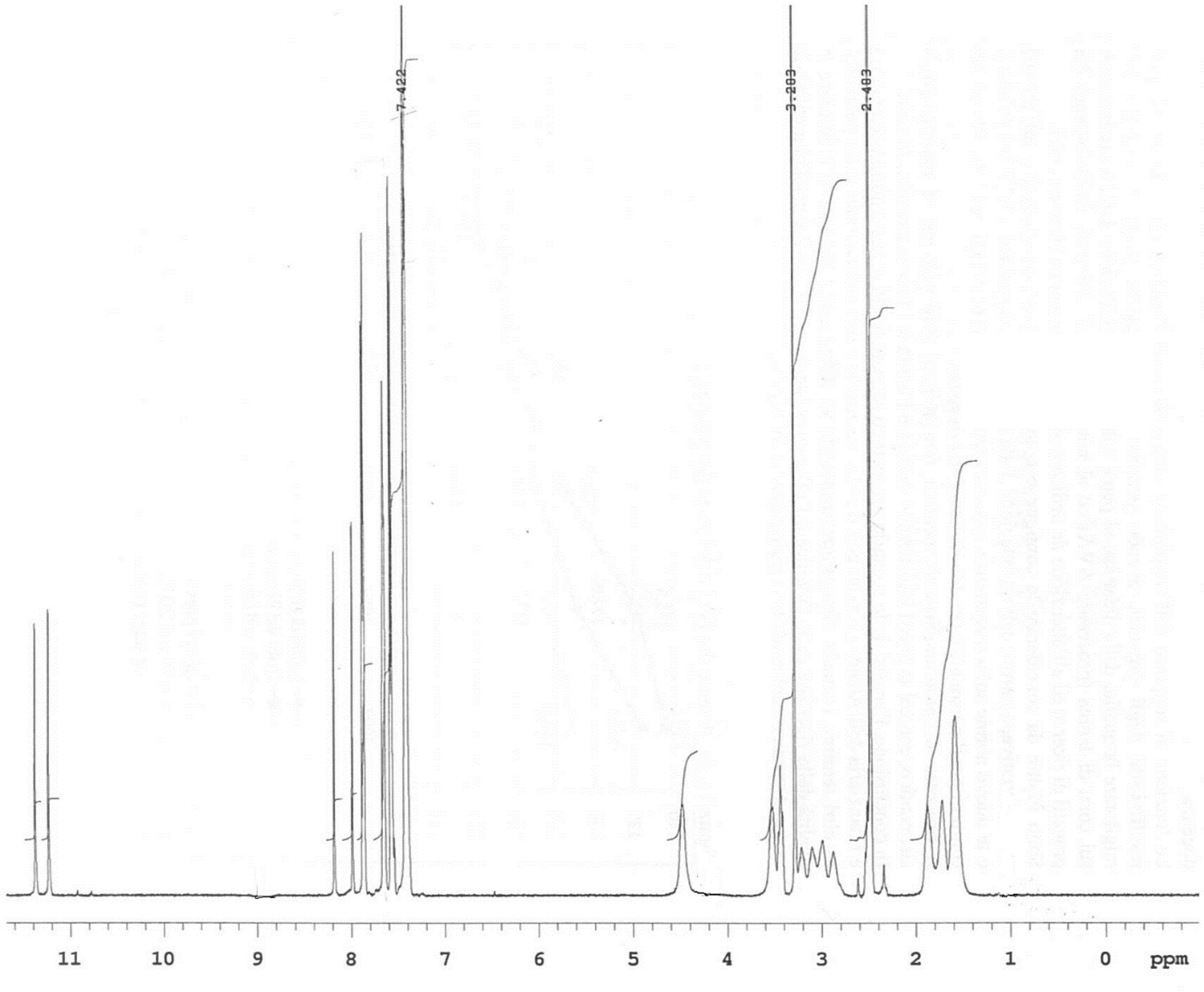

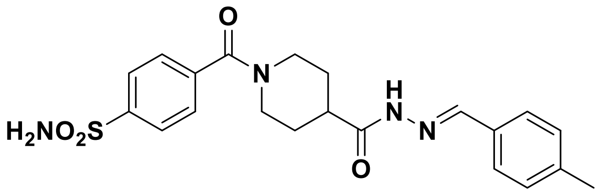

^{1} H NMR, DMSO-d_6, 6

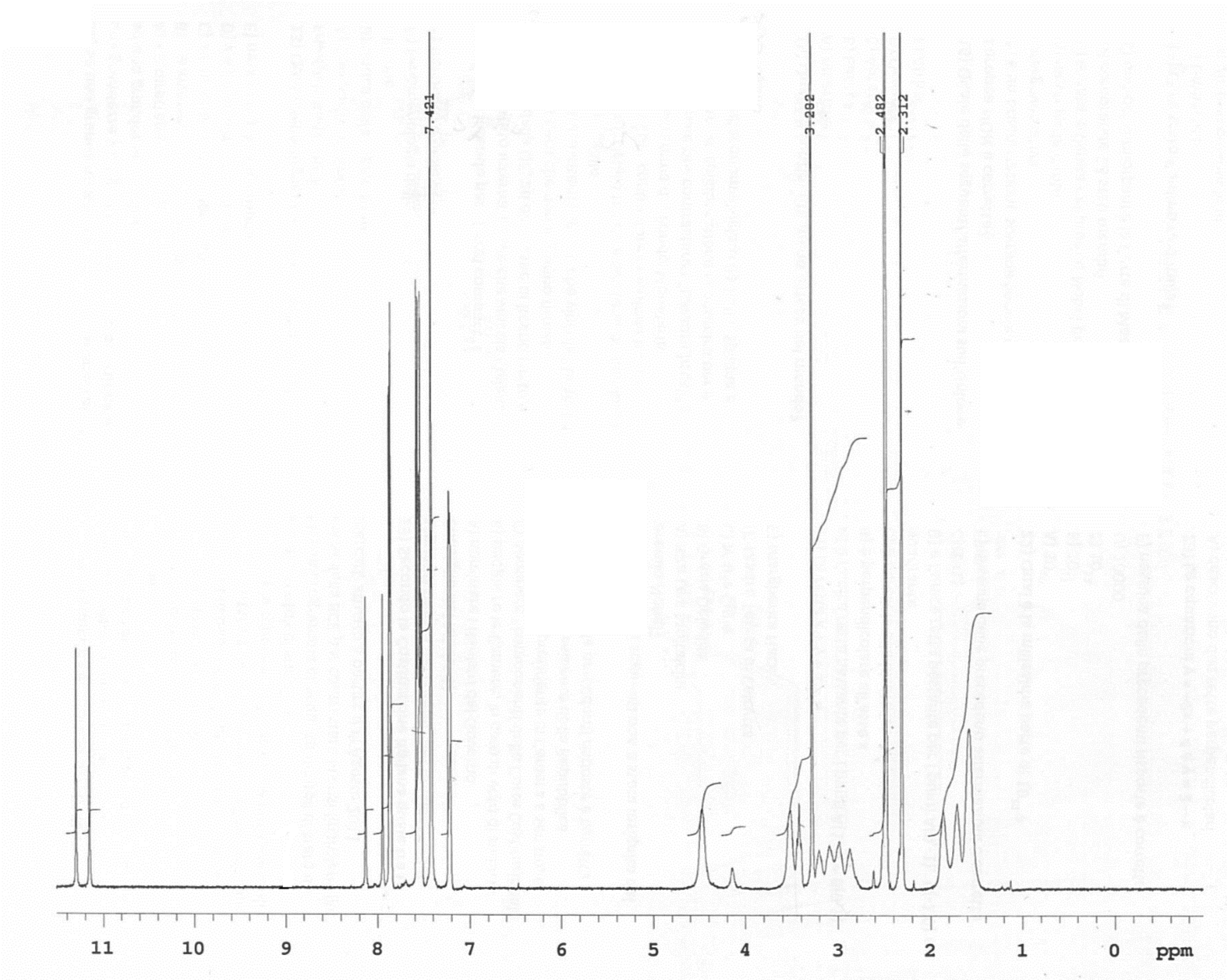

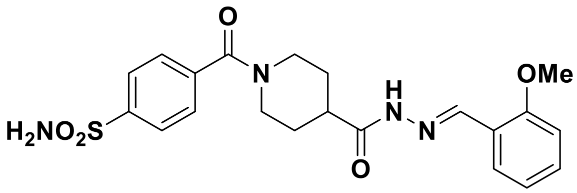

^{1}H NMR, DMSO-d_6, 7

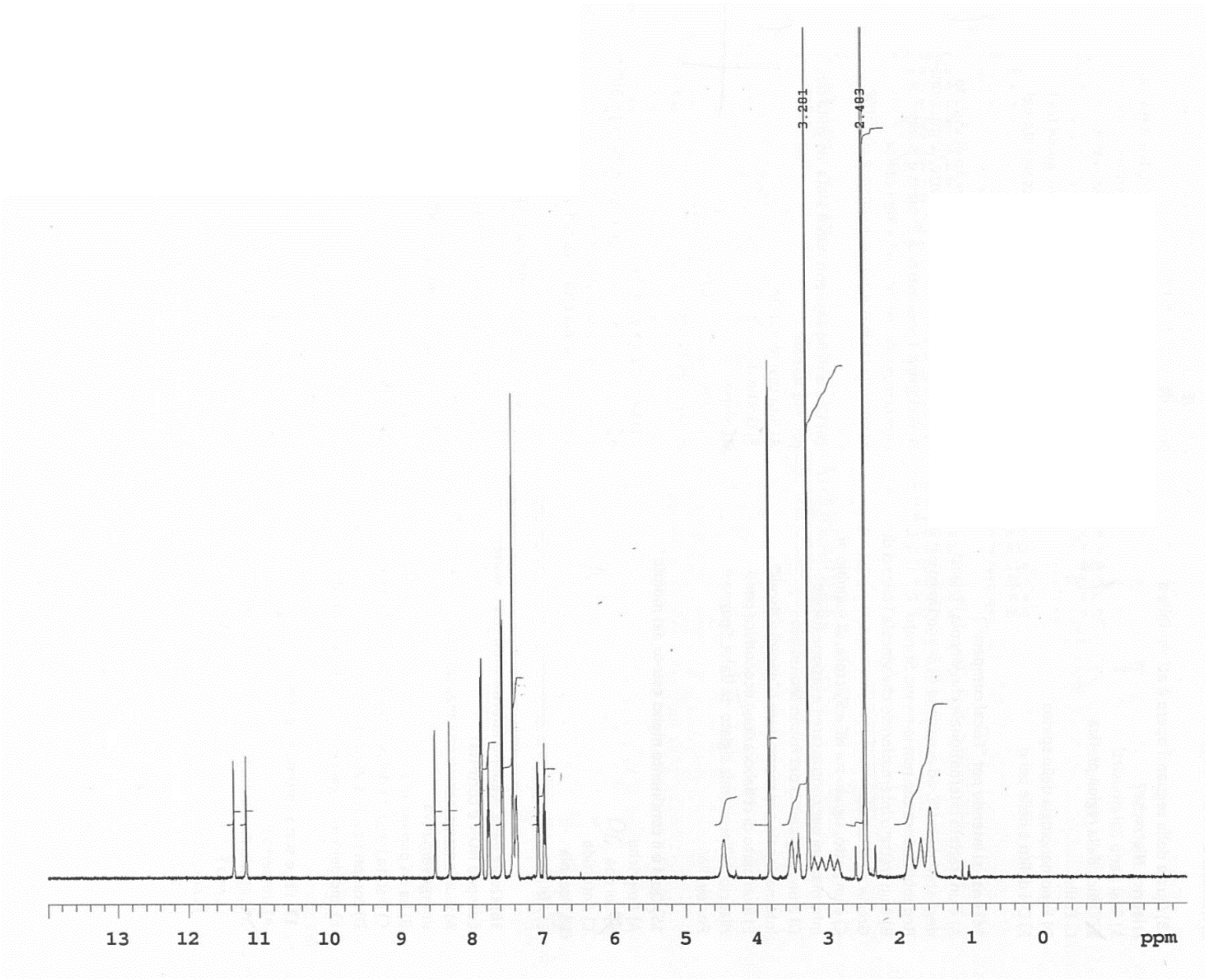

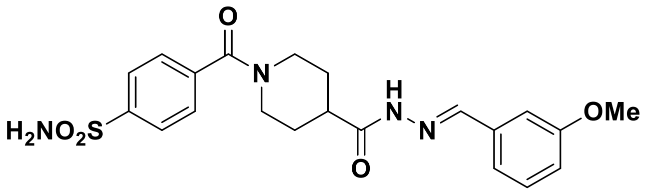

[1] H NMR, DMSO-d_6, 8

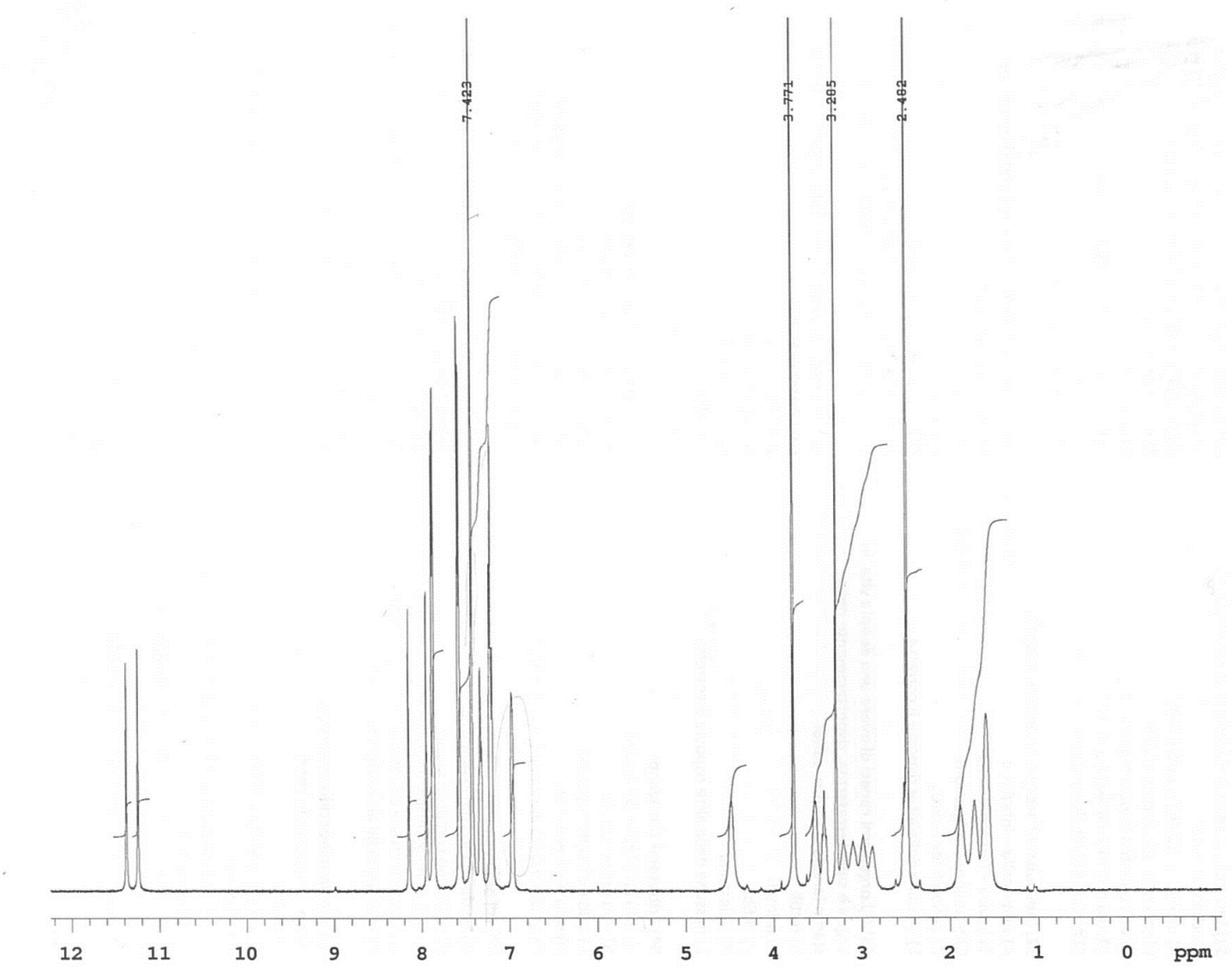

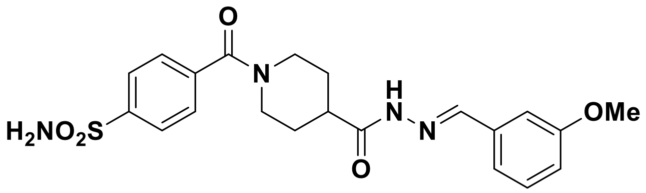

^{13} C NMR, DMSO-d_6, 8

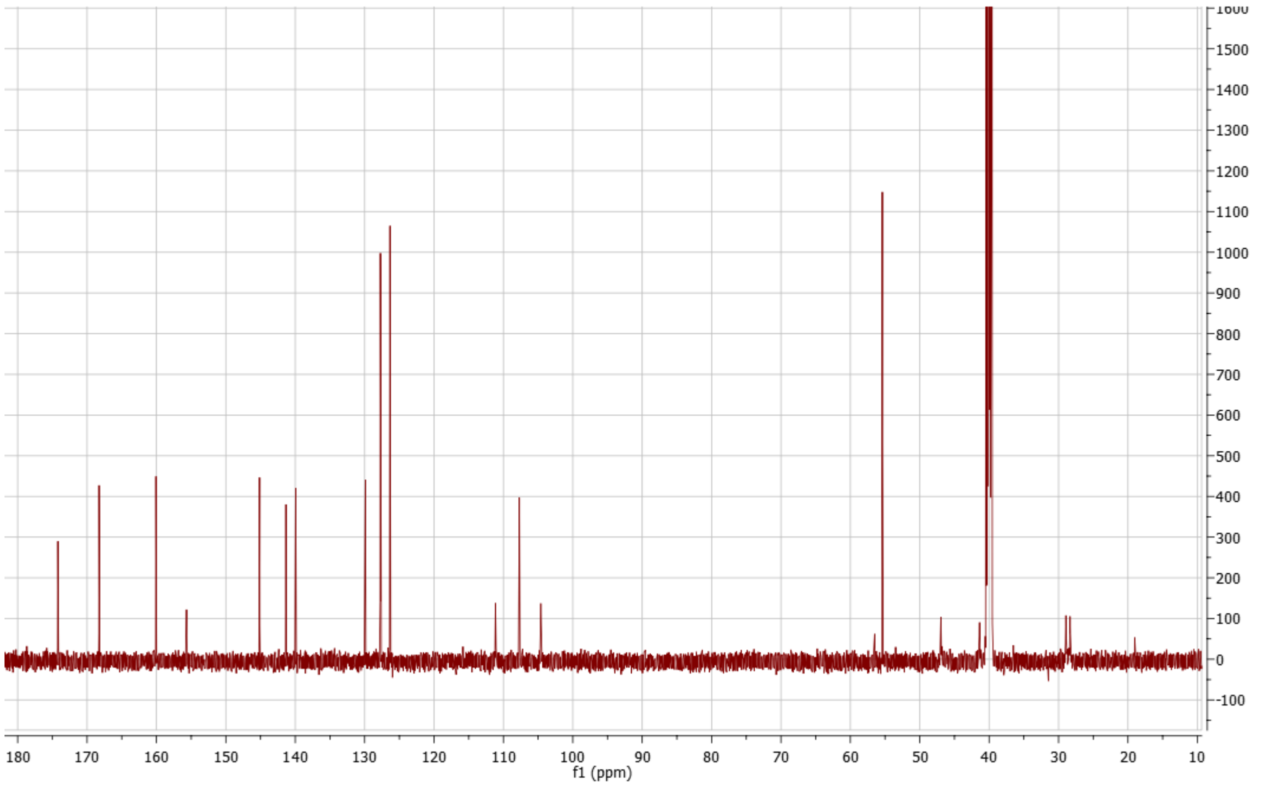

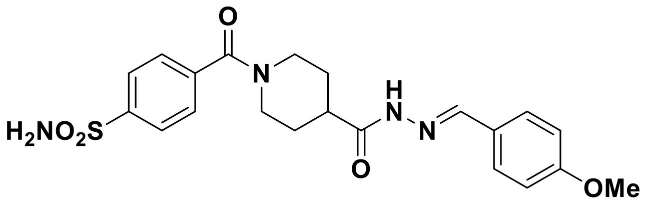

^{1} H NMR, DMSO-*d*$_6$, 9

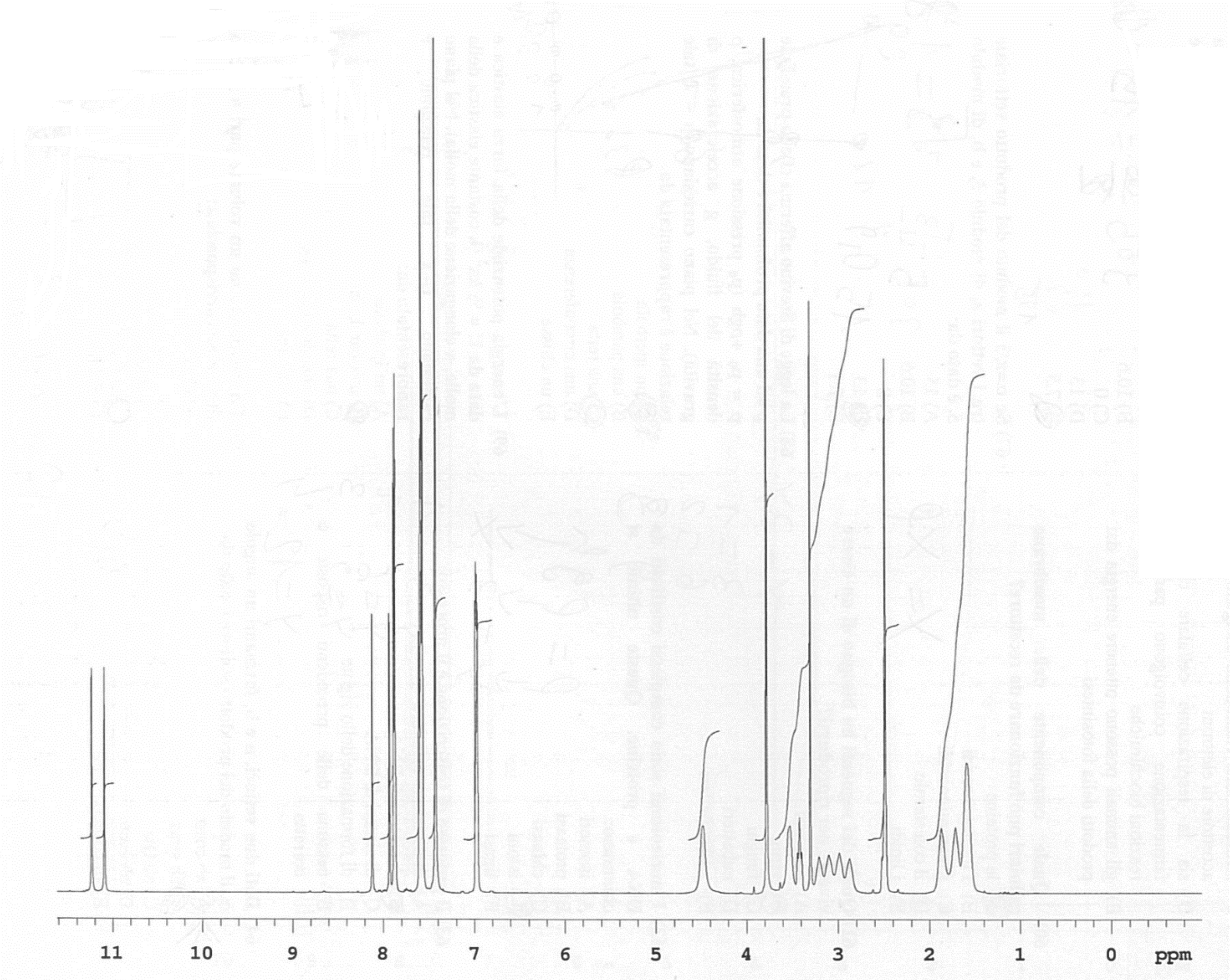

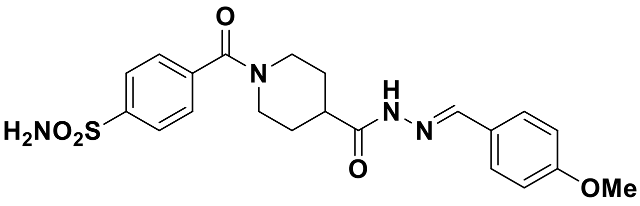

^{13} C NMR, DMSO-d_6, 9

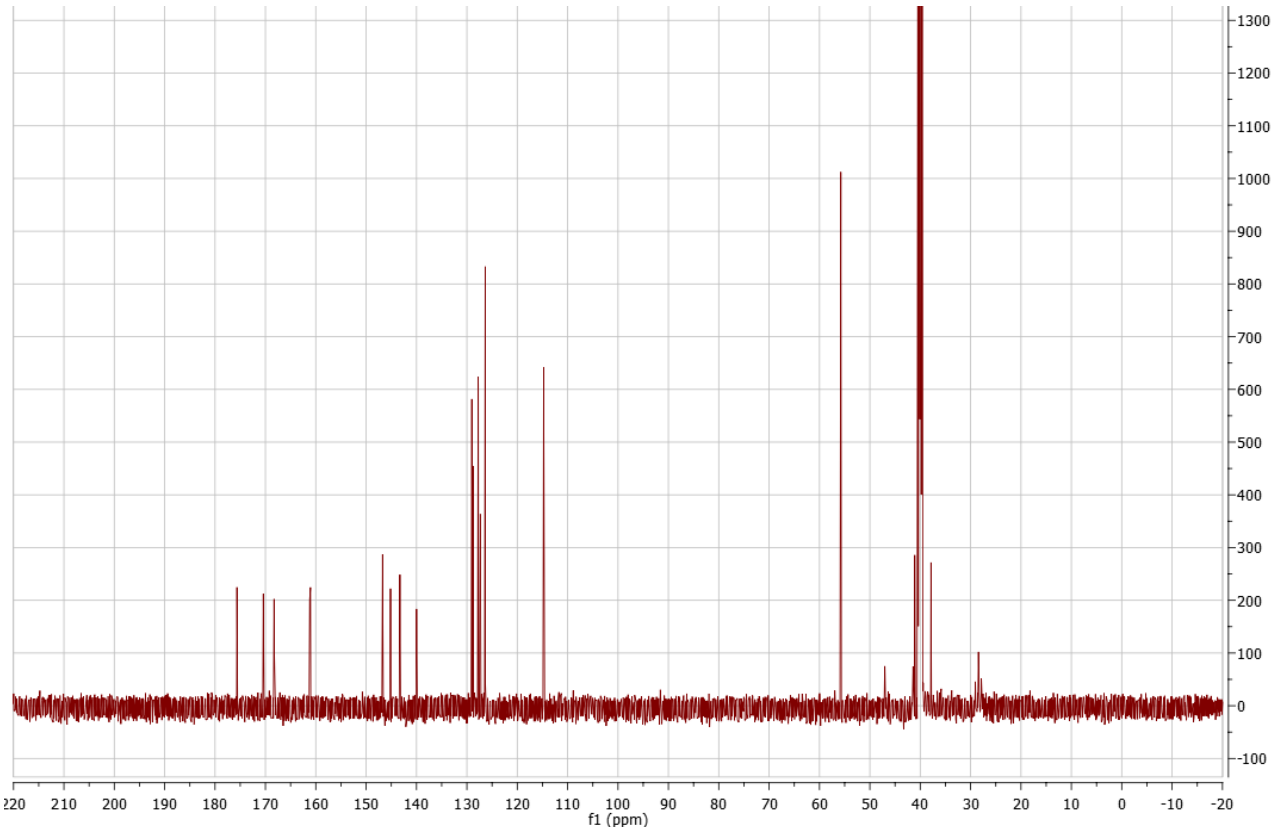

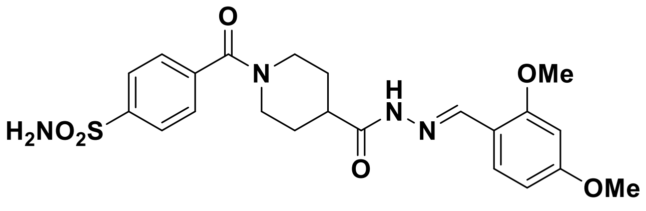

^{1} H NMR, DMSO-d_6, 10

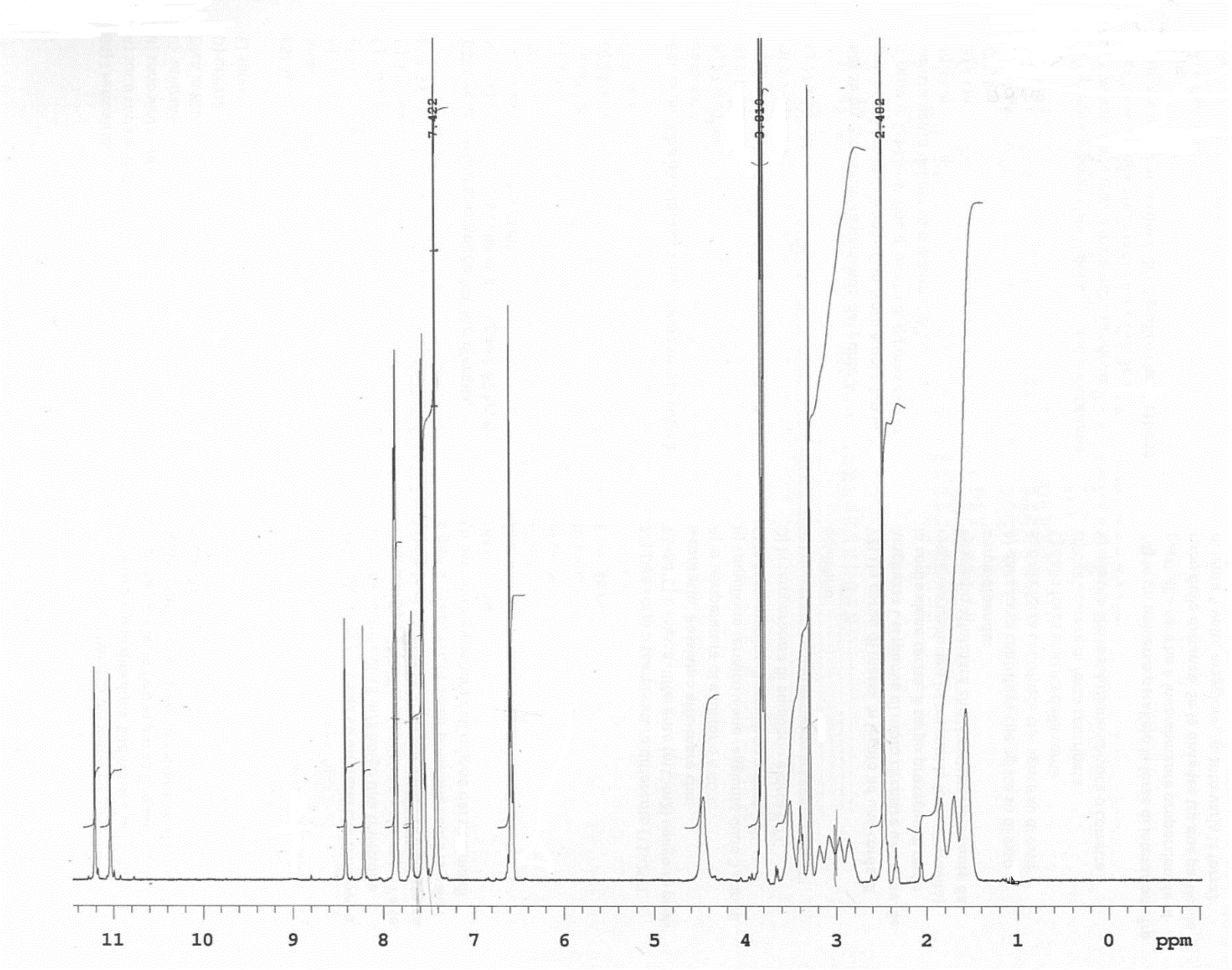

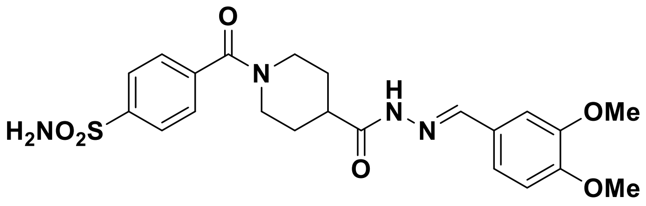

^{1} H NMR, DMSO-d_6, 11

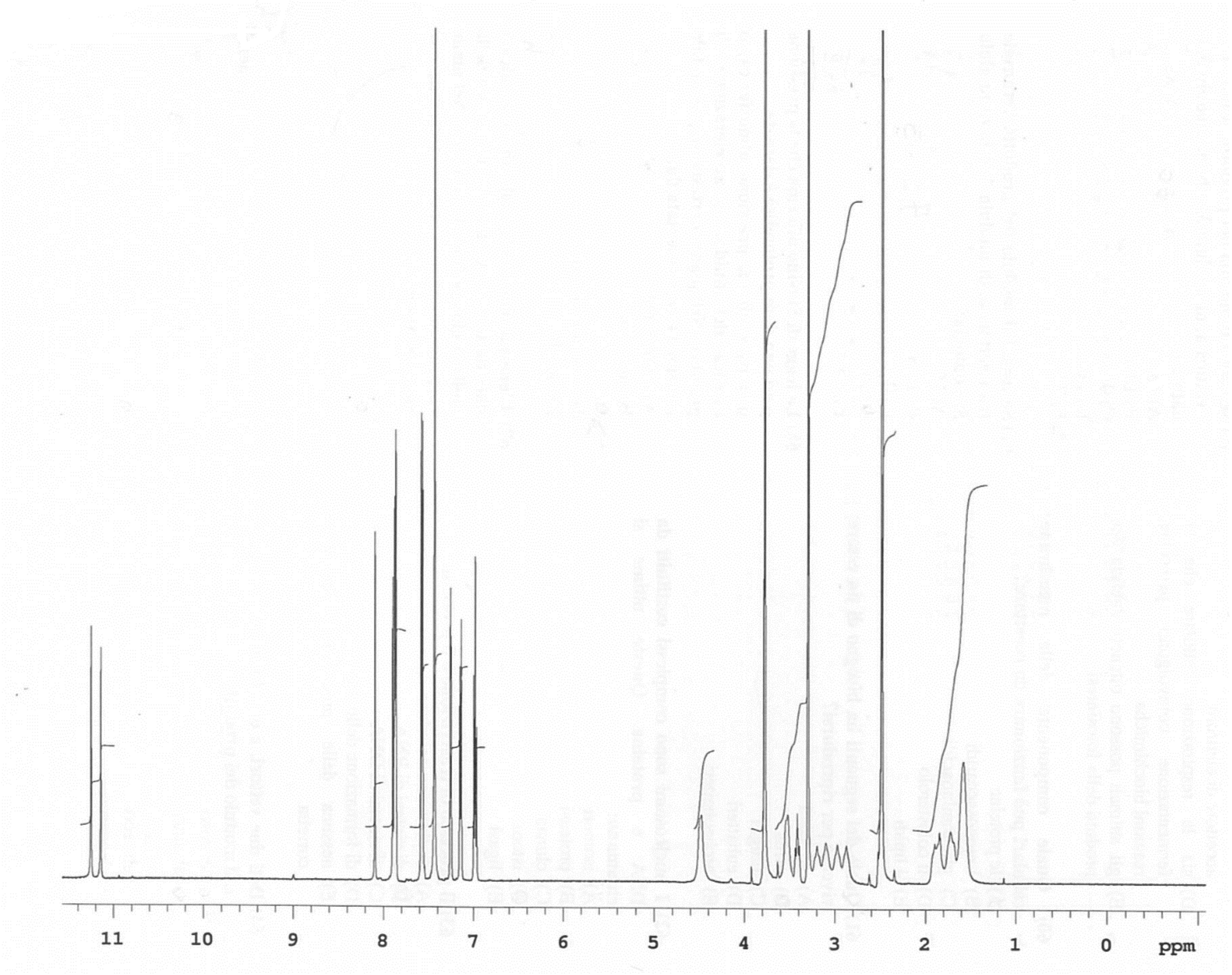

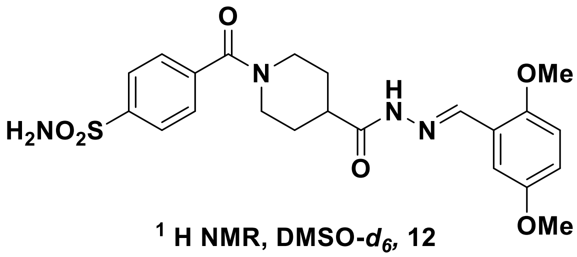

^{1} H NMR, DMSO-d_6, 12

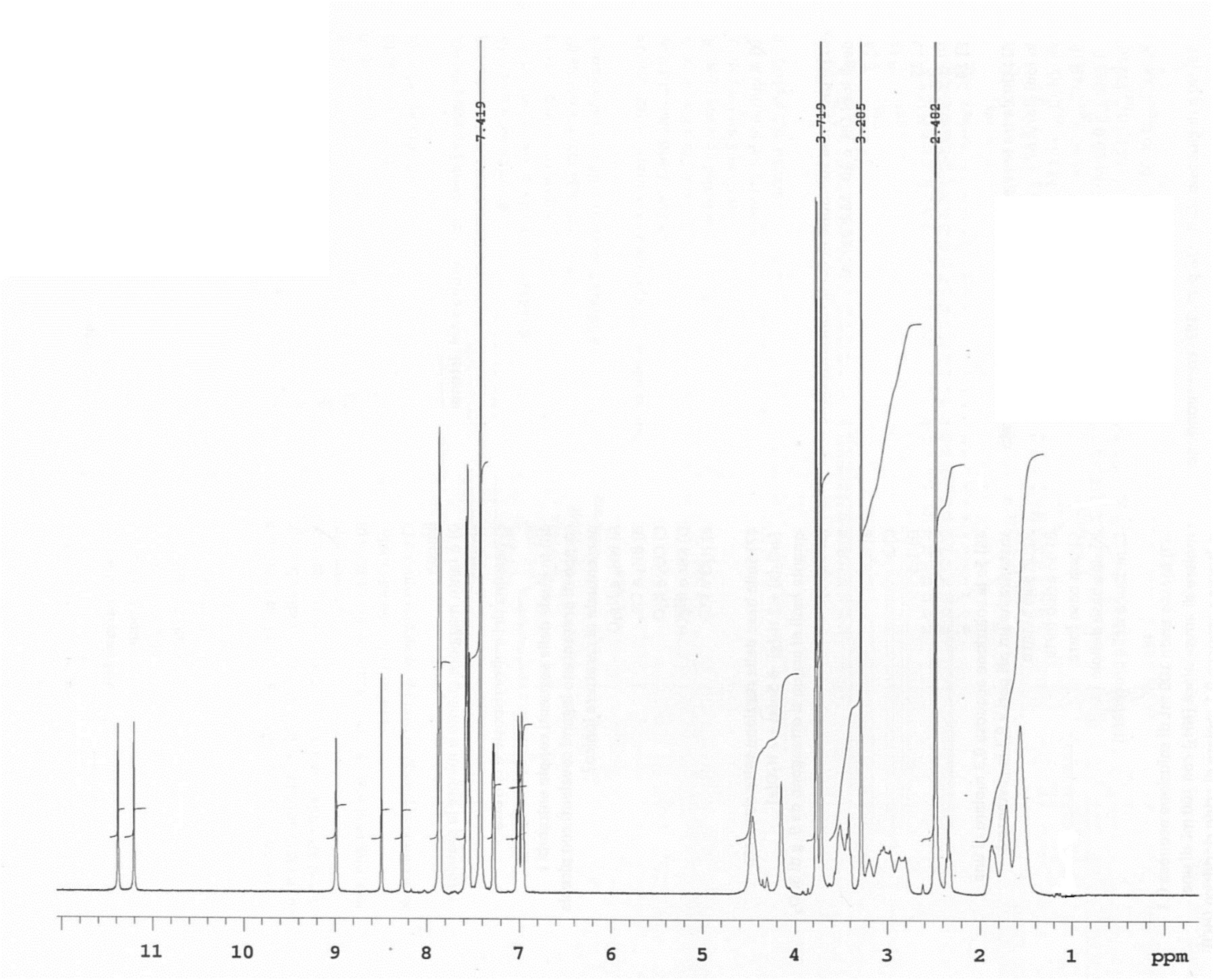

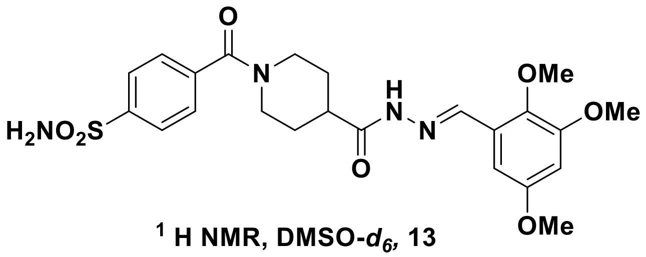

^{1} H NMR, DMSO-d_6, 13

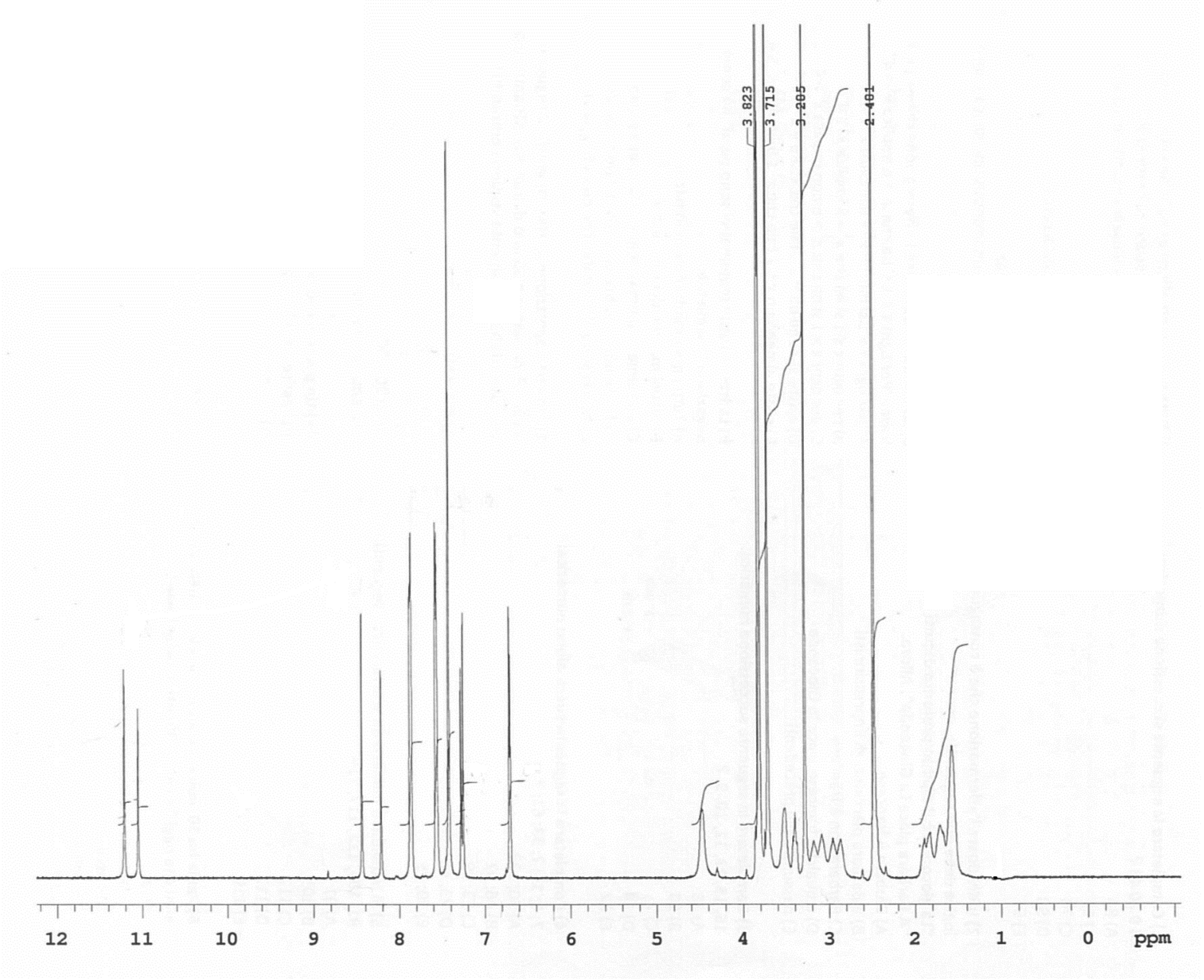

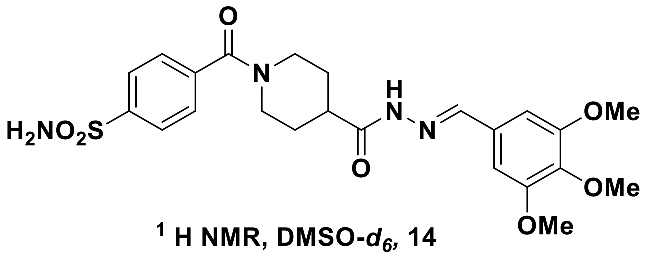

^{1}H NMR, DMSO-*d_6*, 14

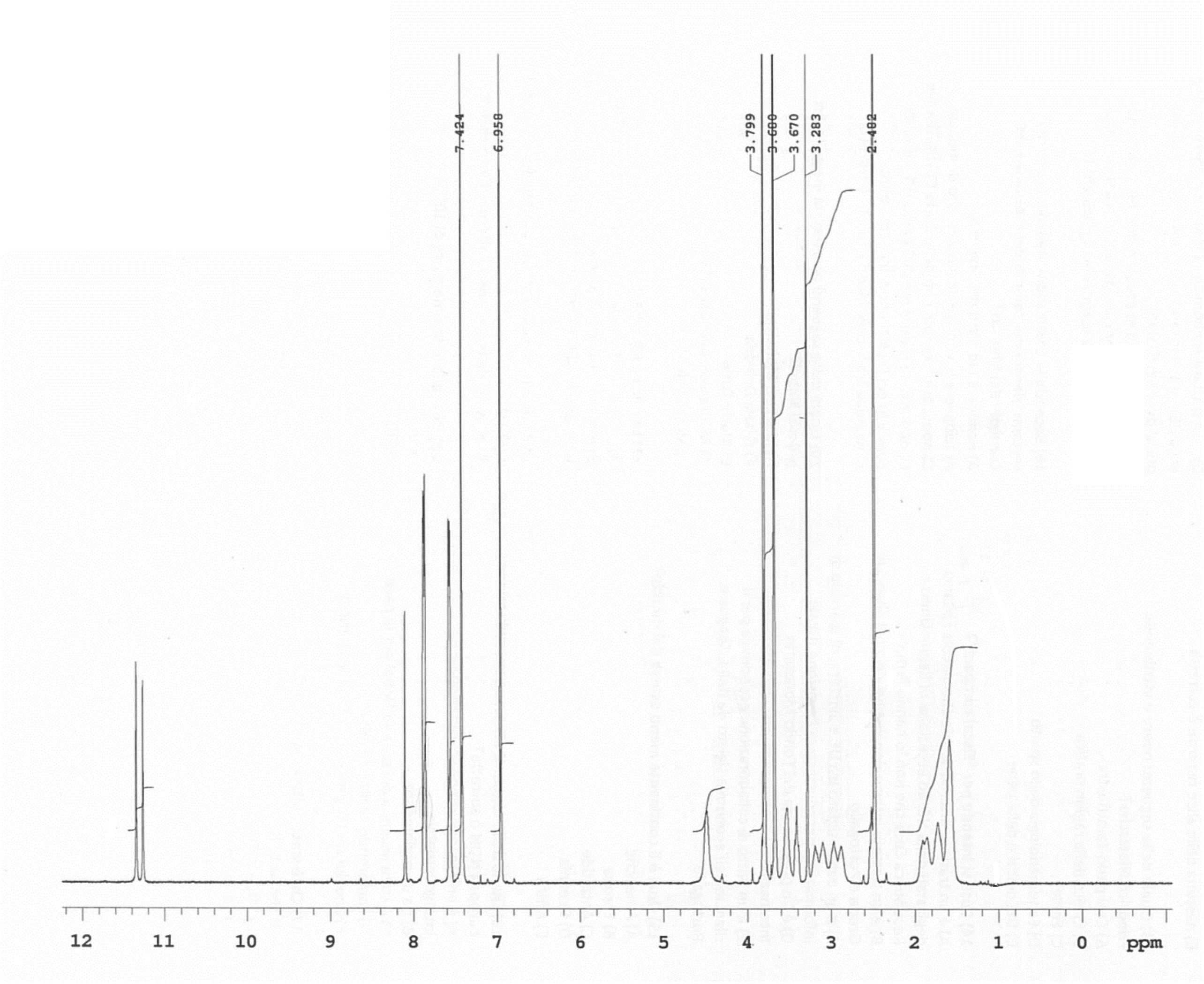

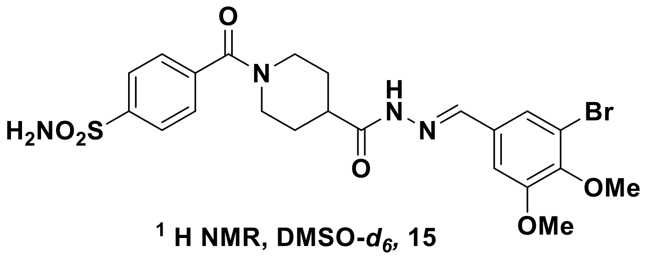

^{1} H NMR, DMSO-d_6, 15

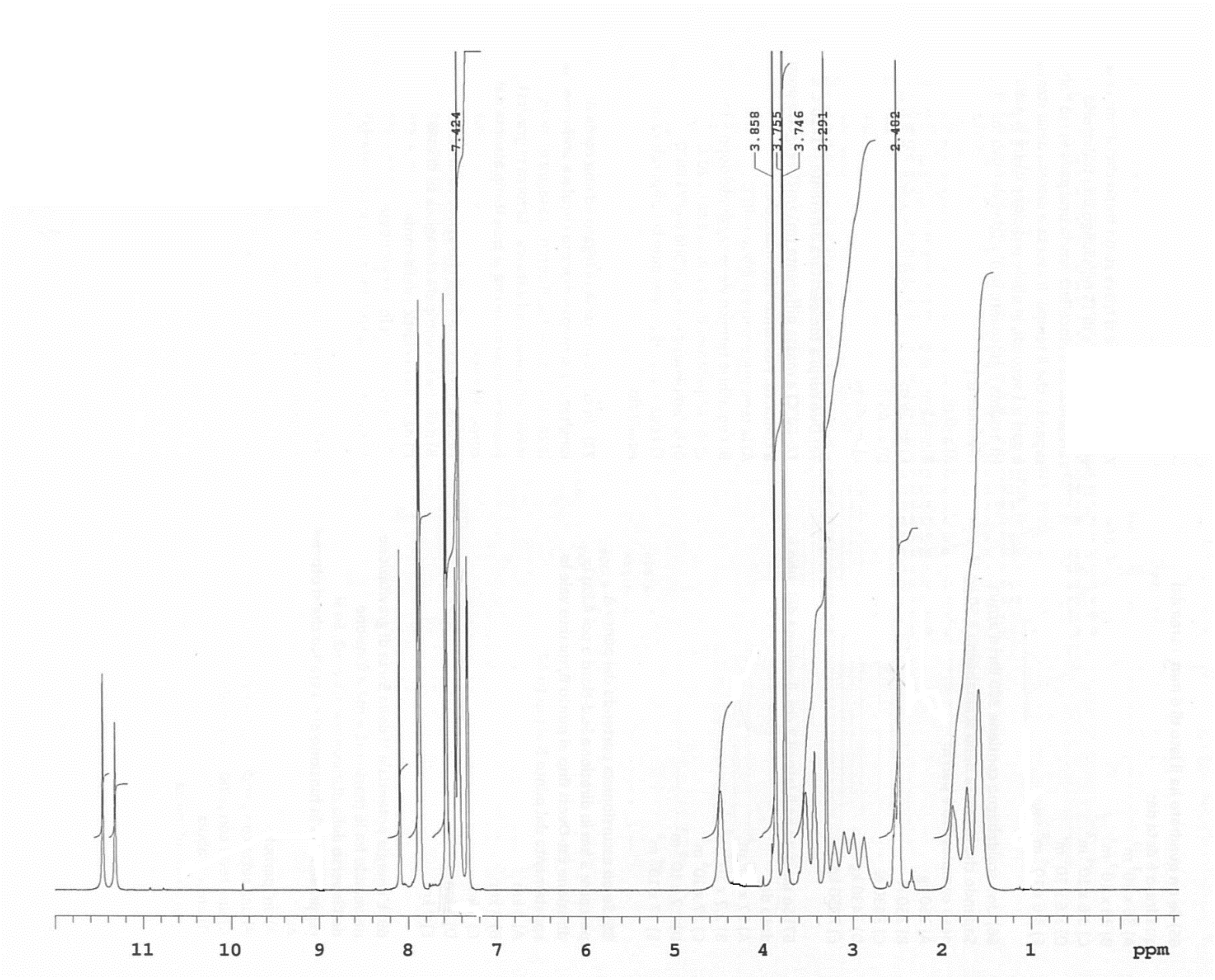

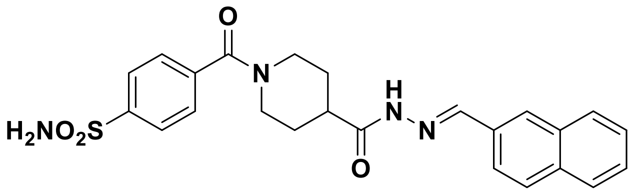

^{1} H NMR, DMSO-*d_6*, 16

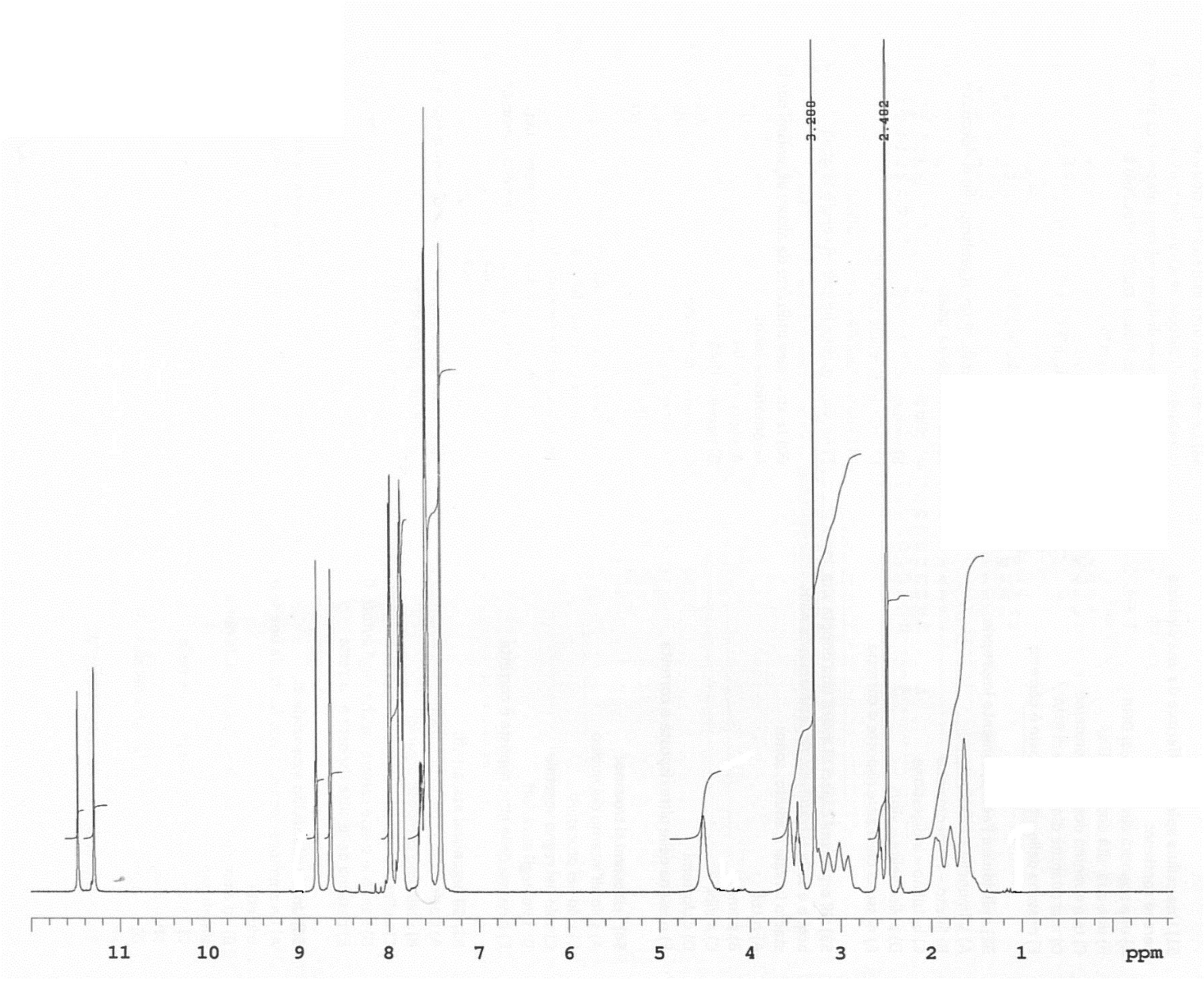

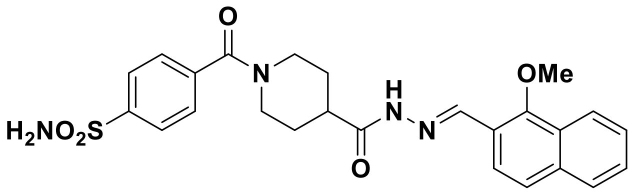

^{1} H NMR, DMSO-d_6, 17

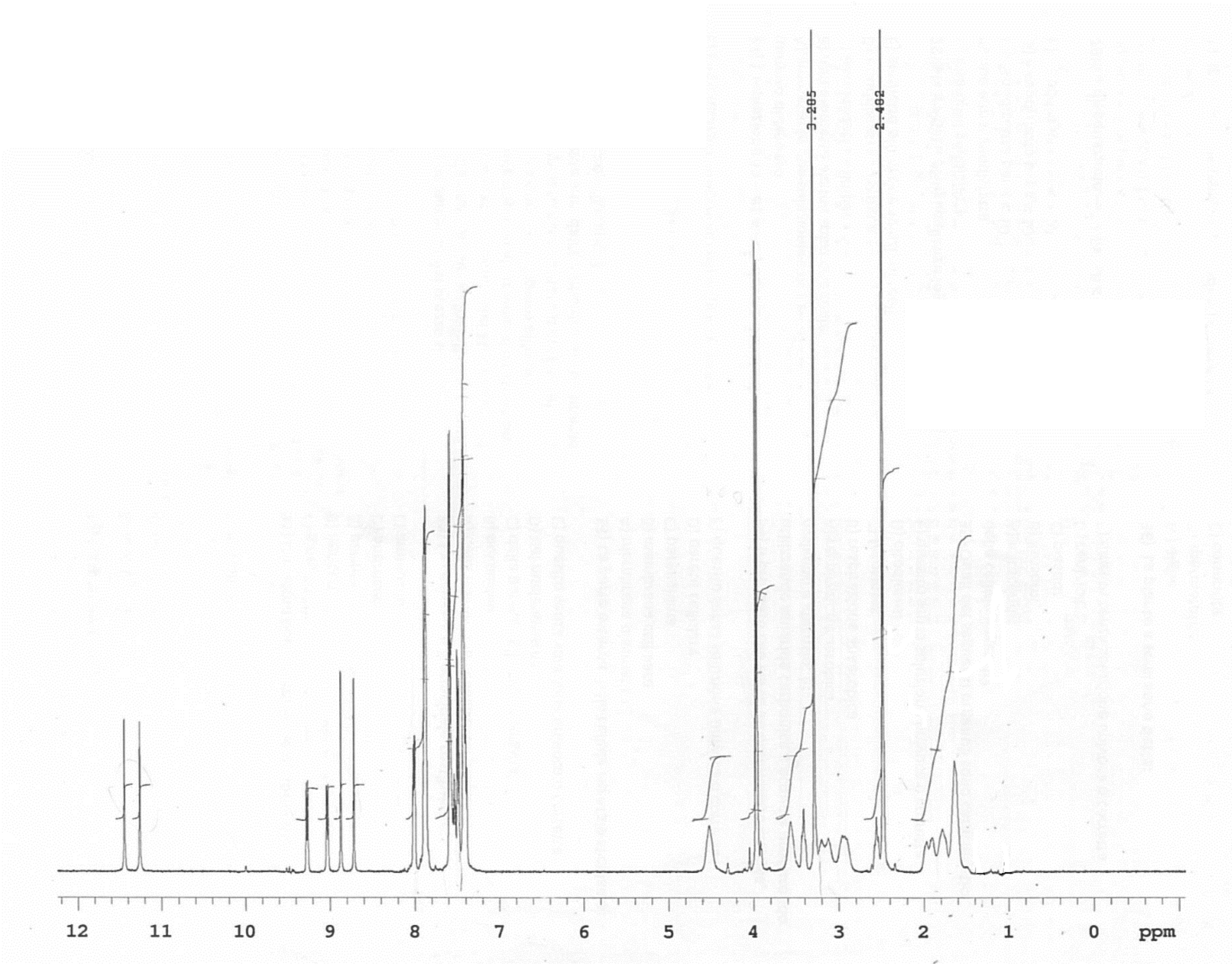

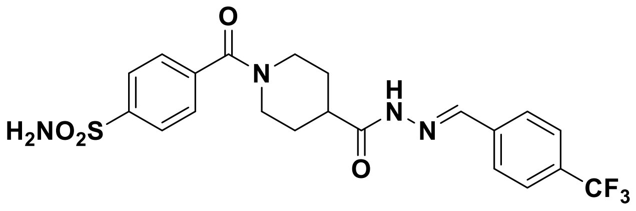

^{1} H NMR, DMSO-d_6, 18

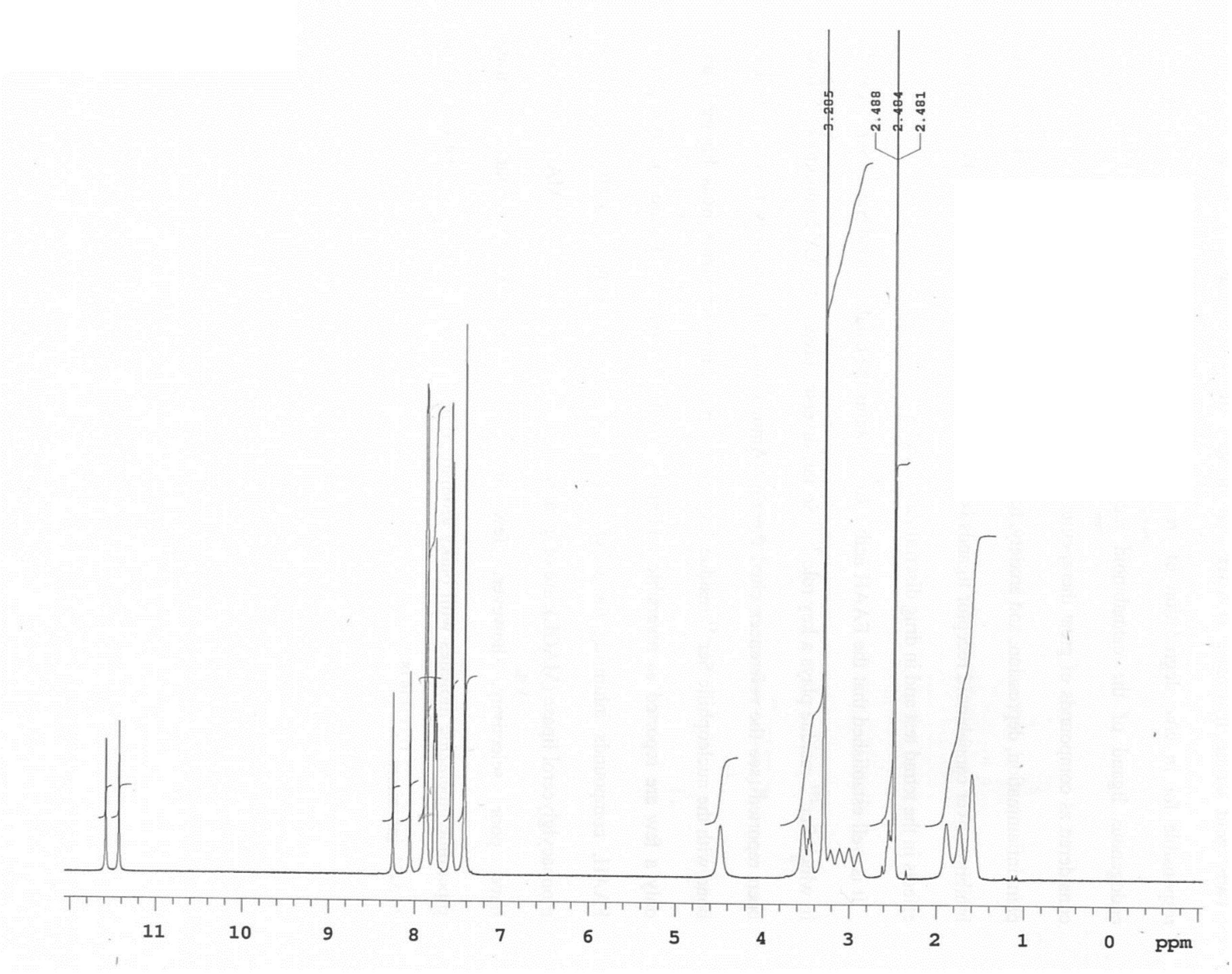

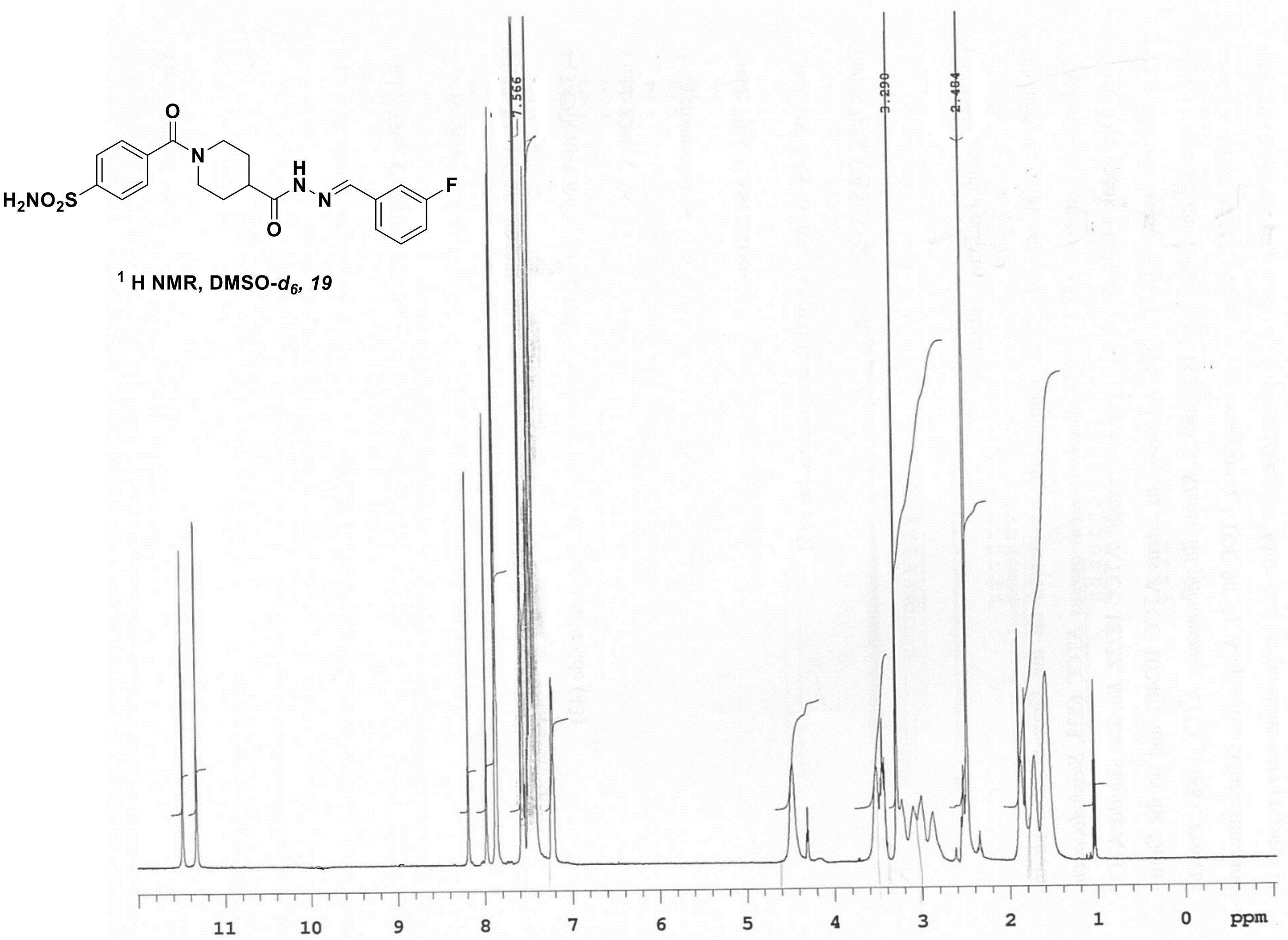

^{1}H NMR, DMSO-d_6, *19*

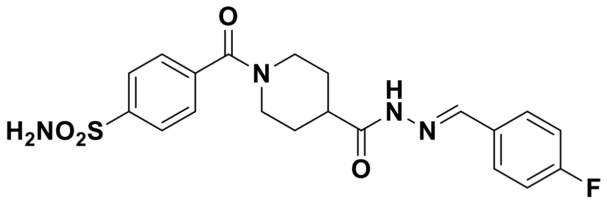

^{1} H NMR, DMSO-d_6, 20

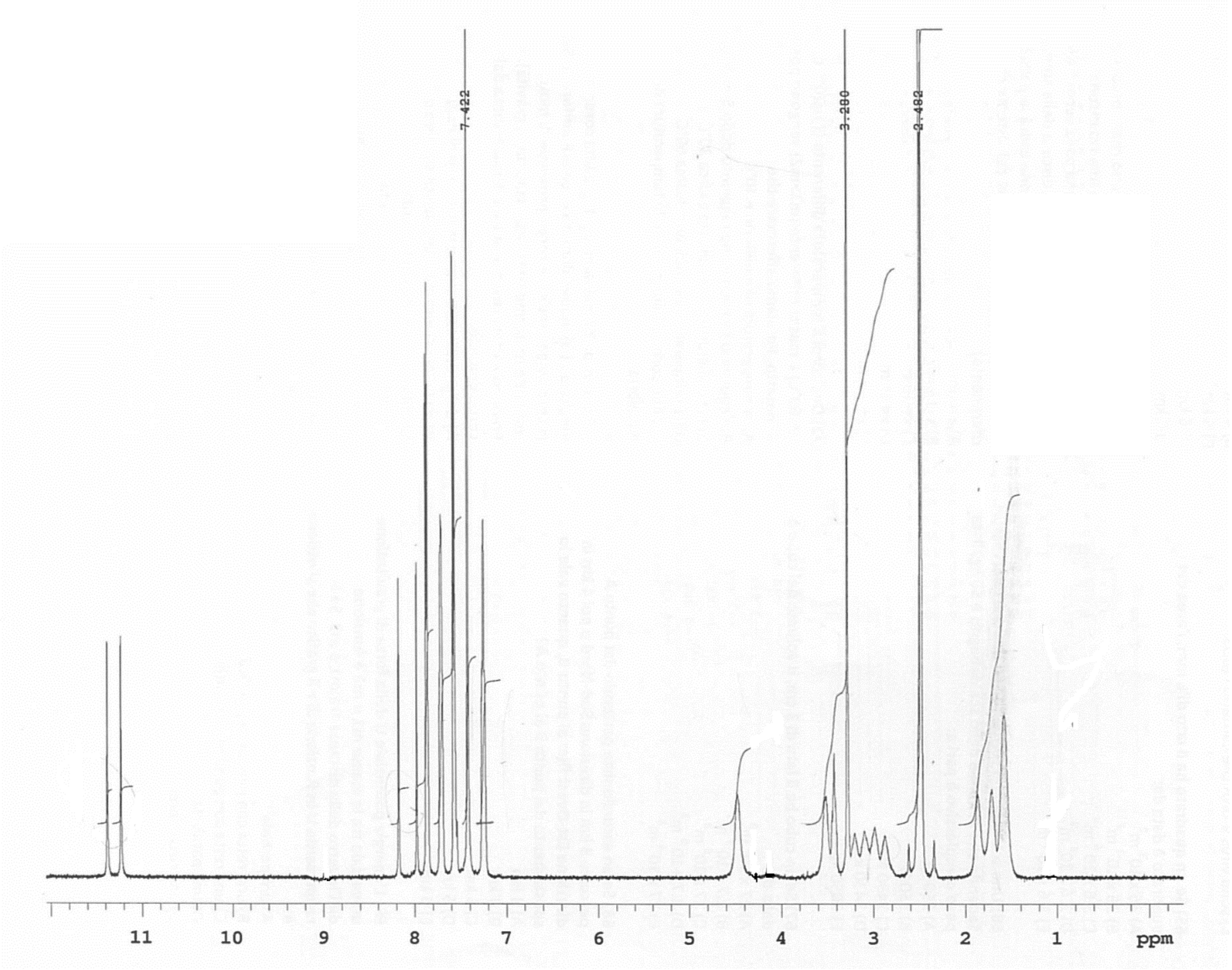

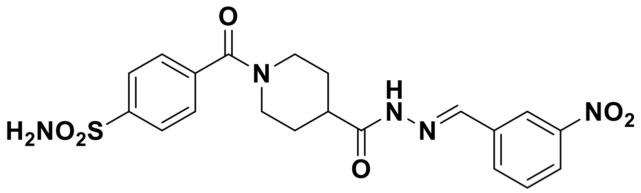

^{1} H NMR, DMSO-d_6, 21

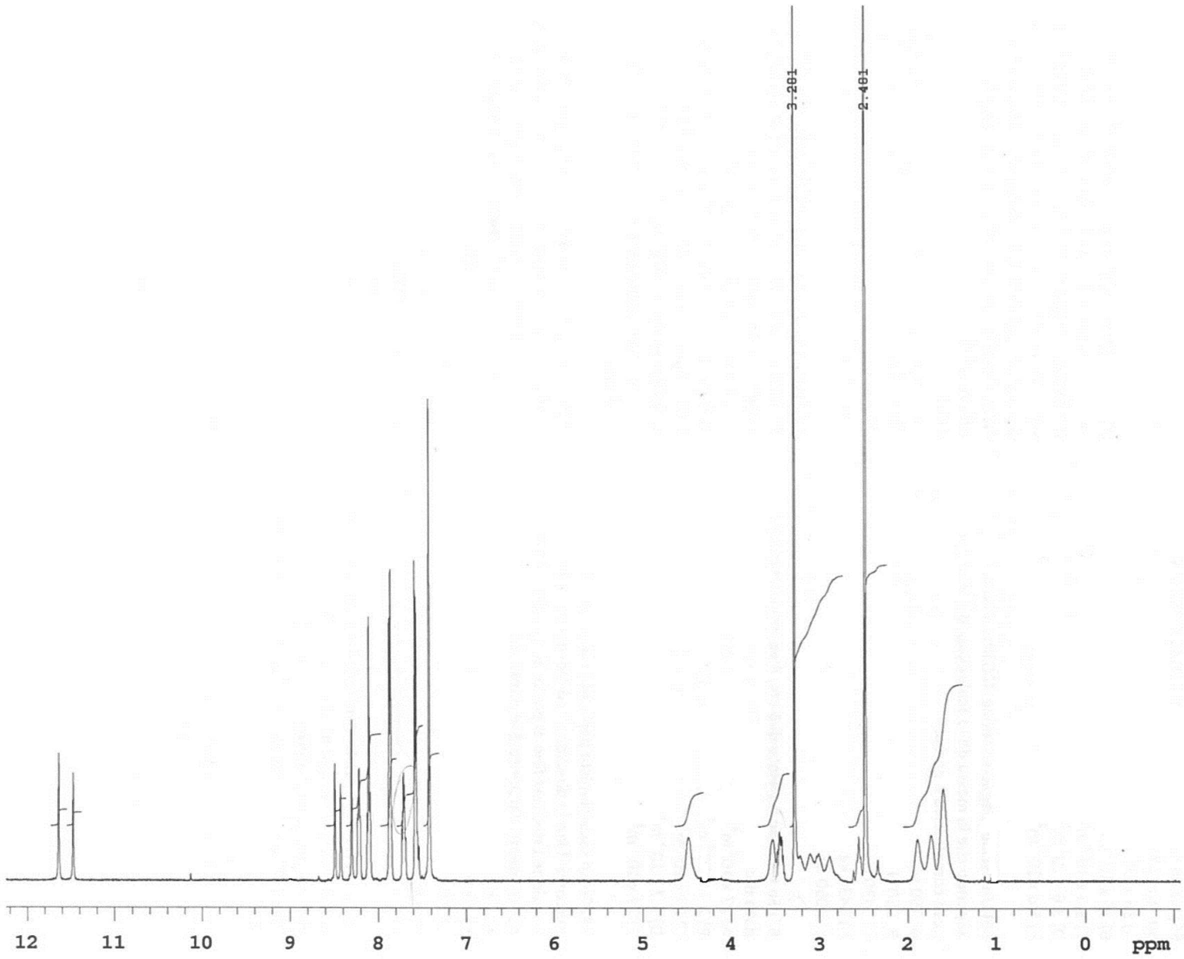

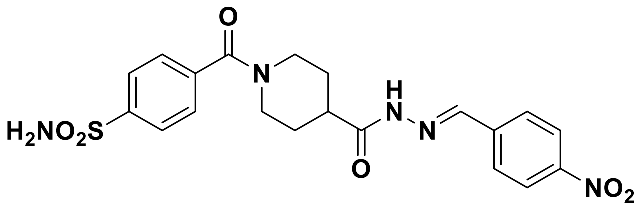

^{1} H NMR, DMSO-d_6, 22

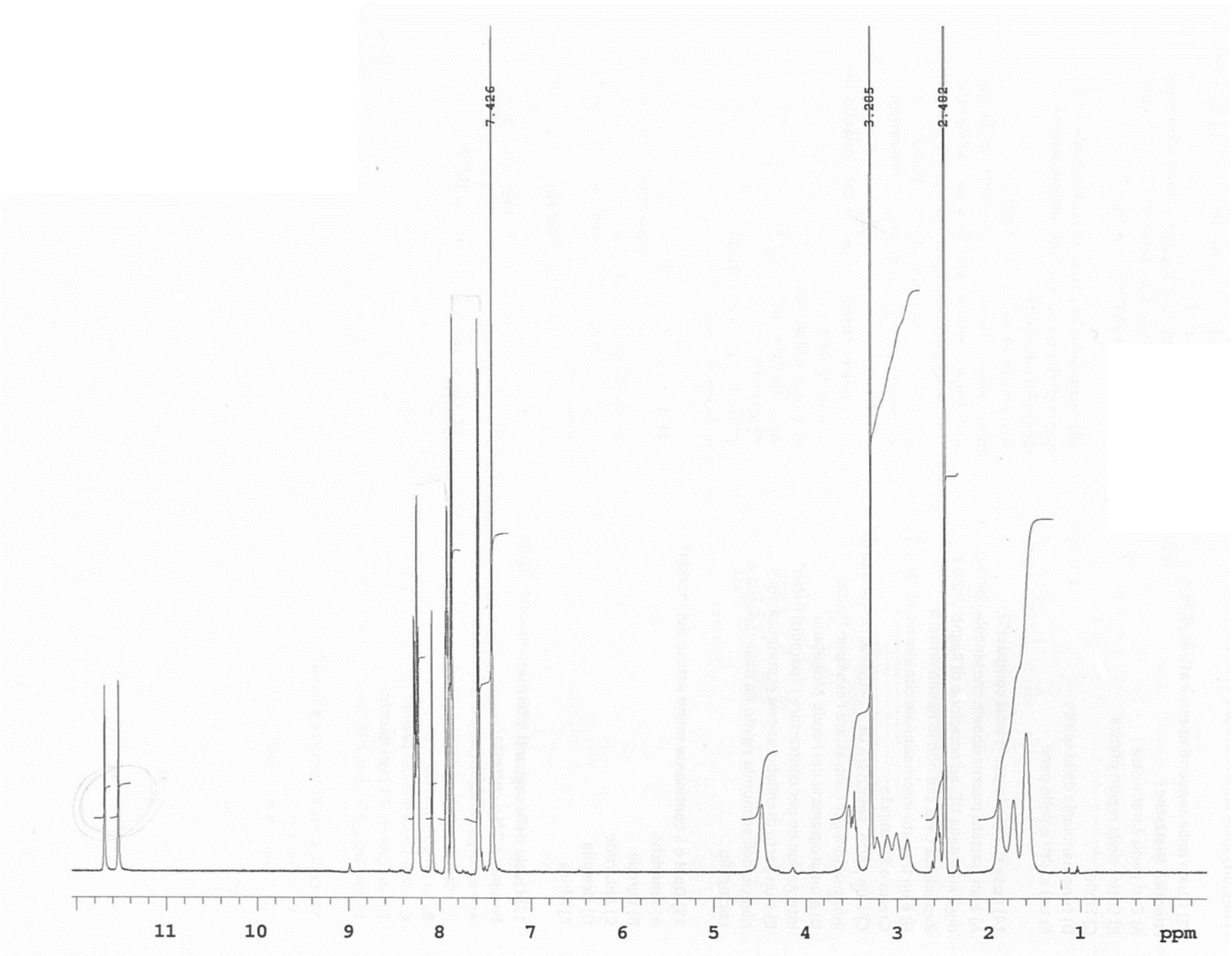

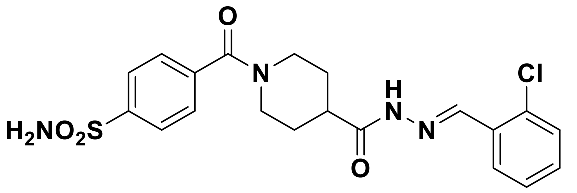

[1] H NMR, DMSO-d_6, 23

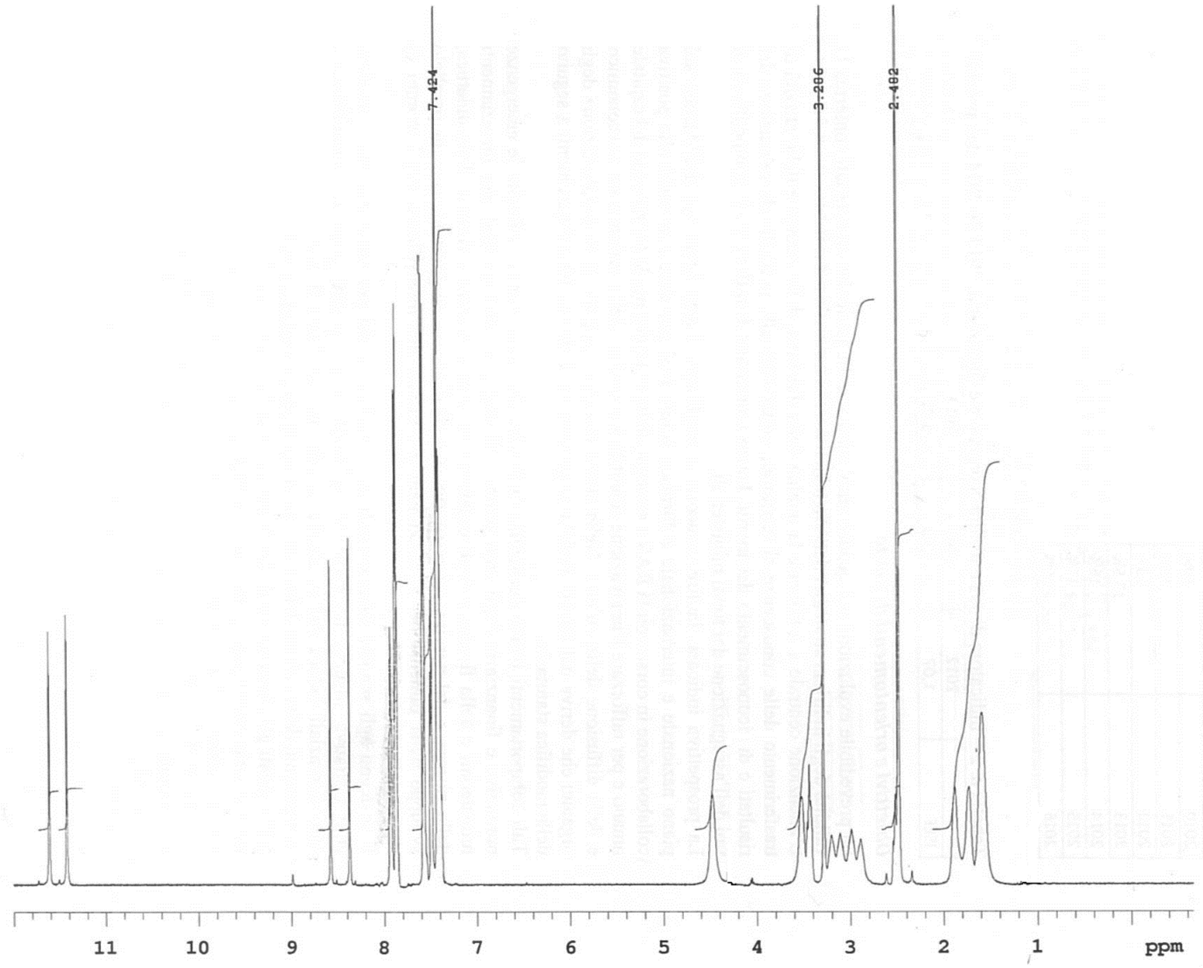

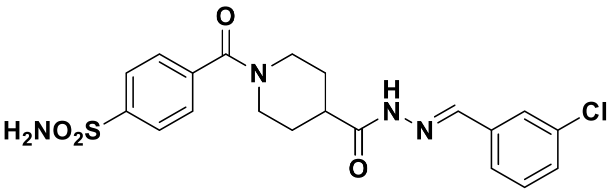

^{1} H NMR, DMSO-d_6, 24

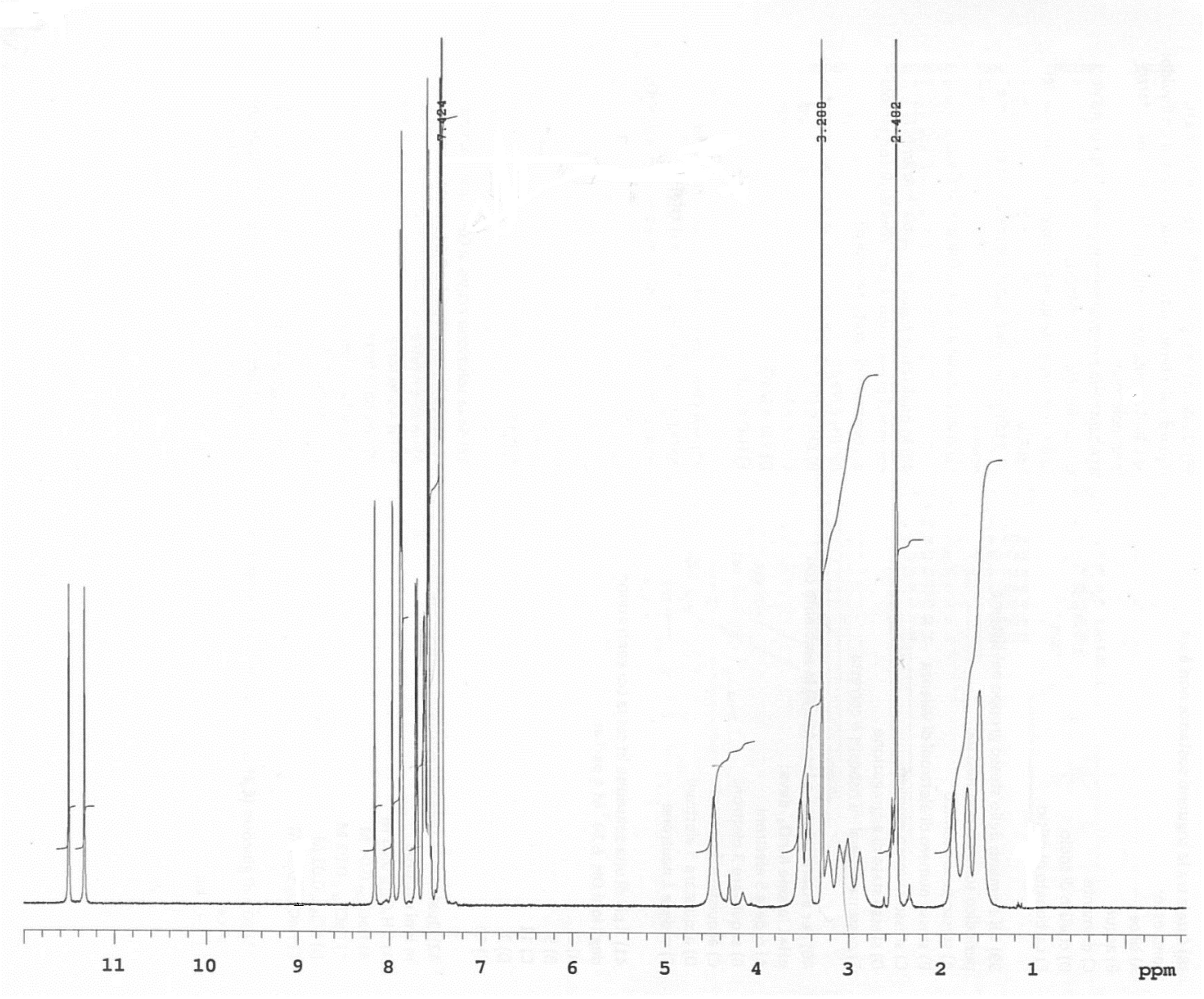

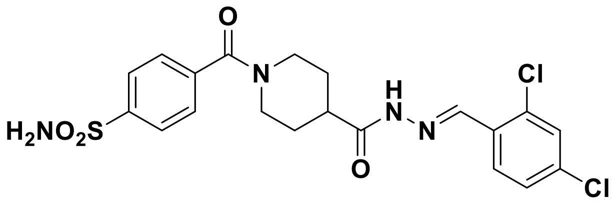

[1] H NMR, DMSO-d_6, 25

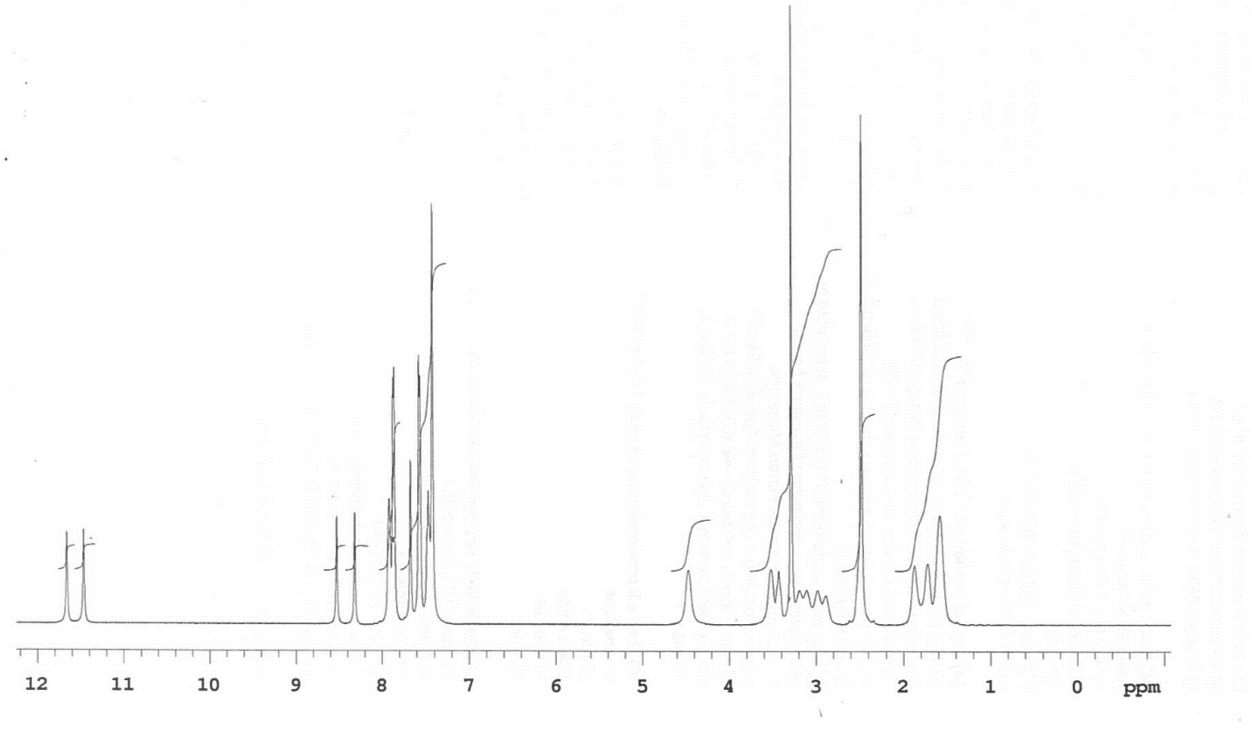

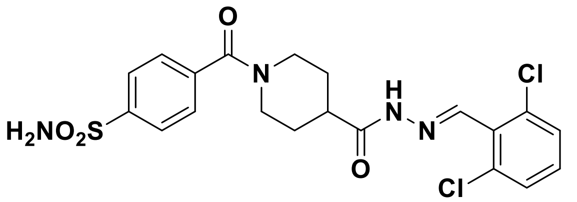

^{1} H NMR, DMSO-d_6, 26

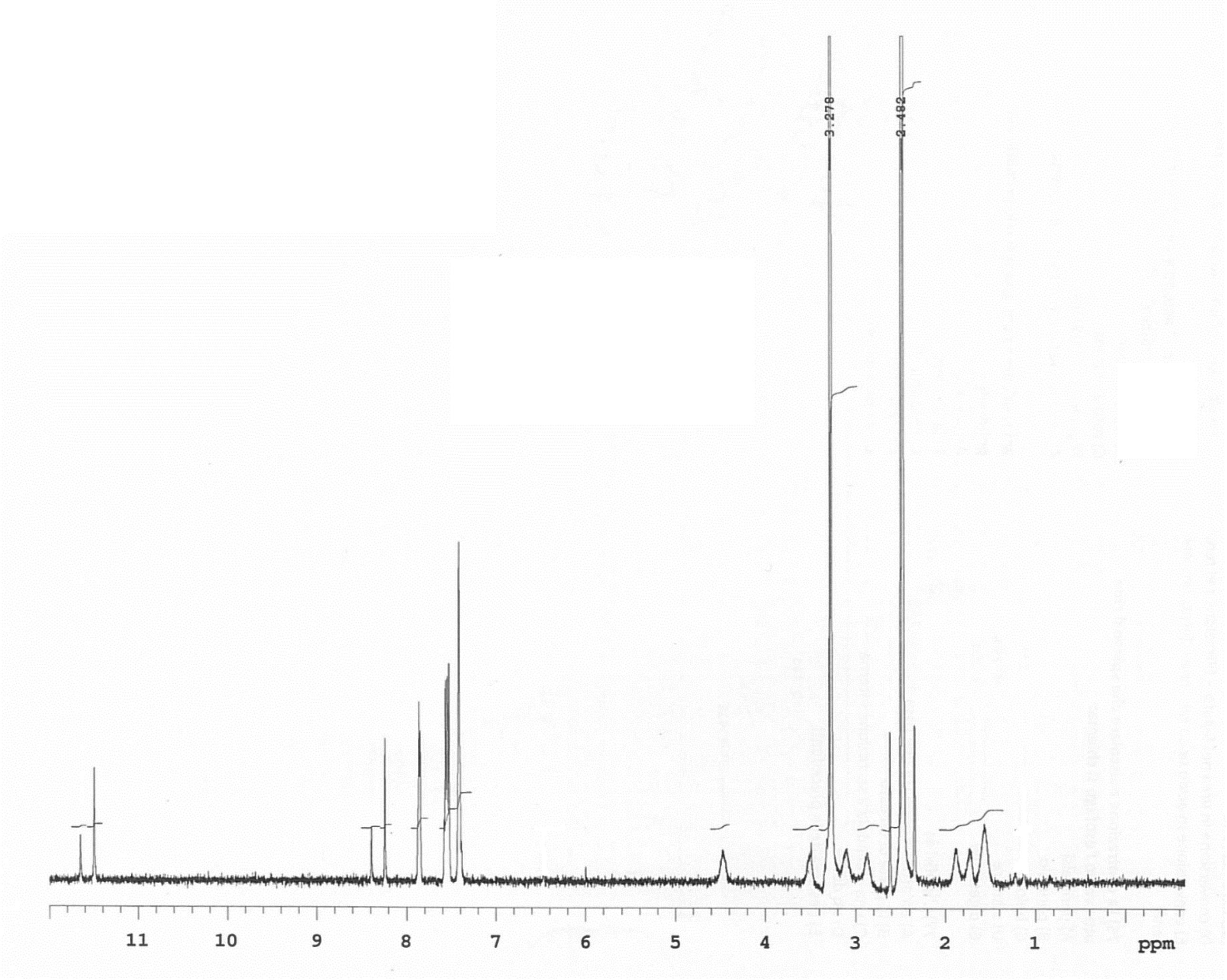

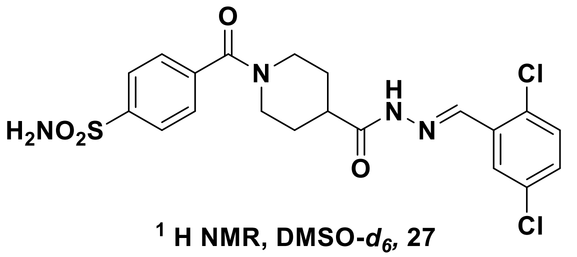

1 H NMR, DMSO-d_6, 27

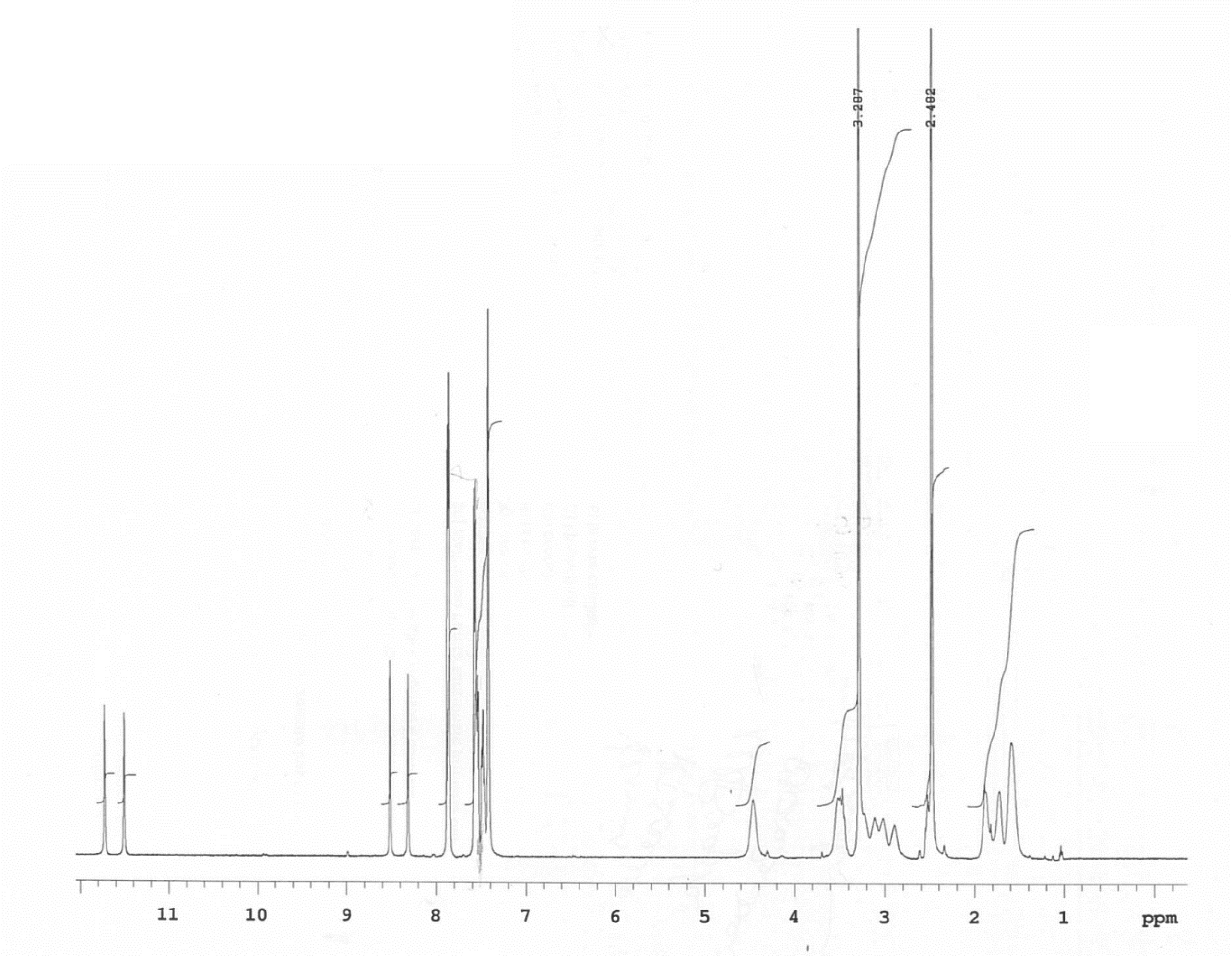

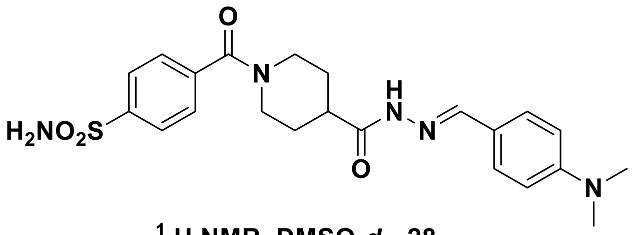

^{1}H NMR, DMSO-d_6, 28

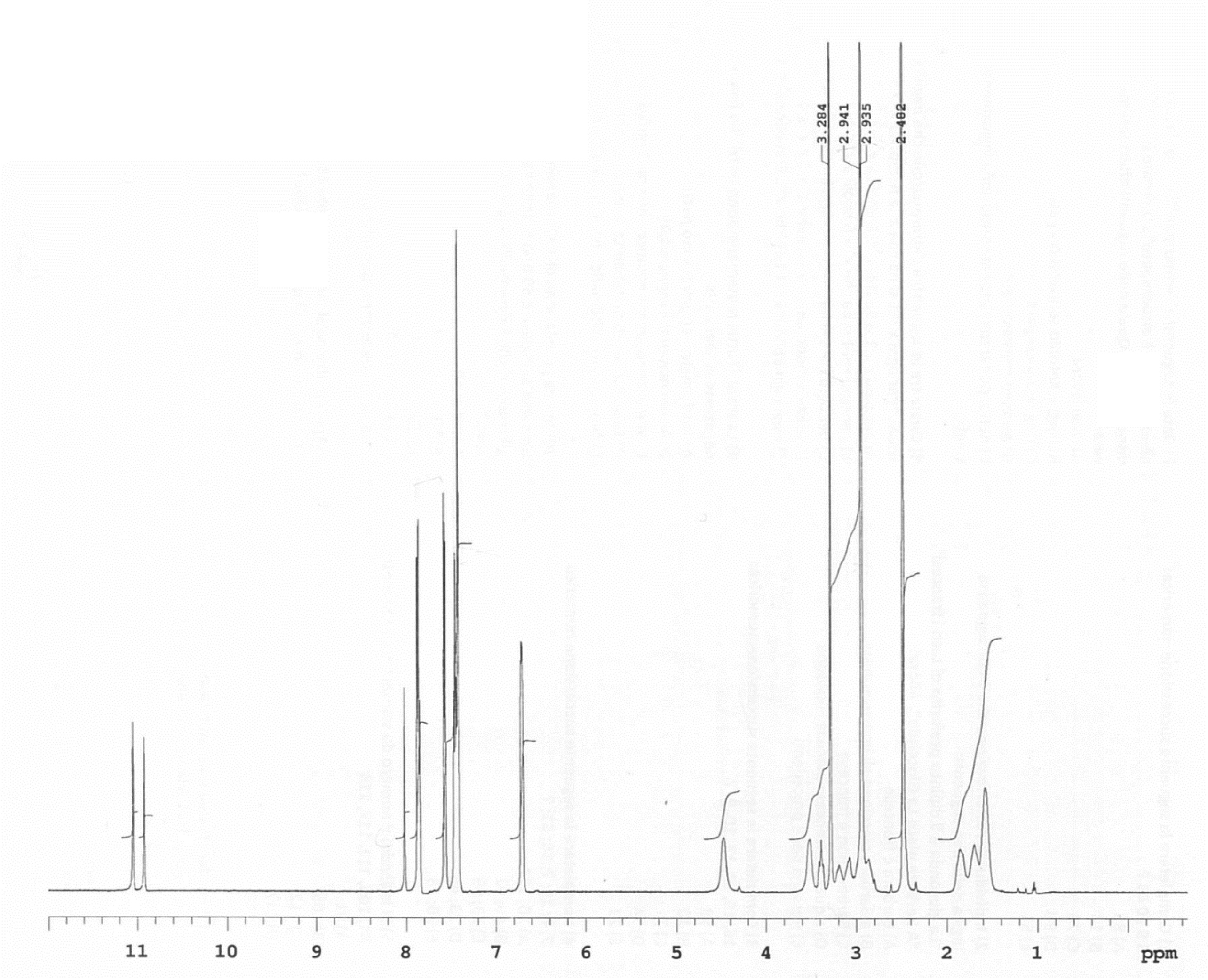

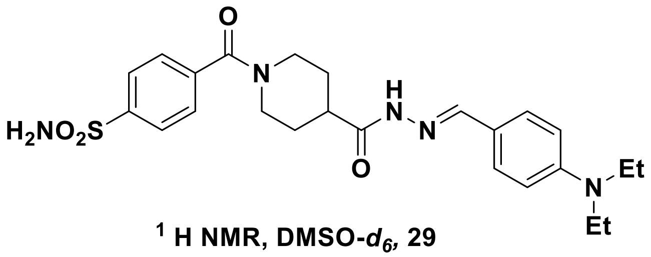

^{1}H NMR, DMSO-d_6, 29

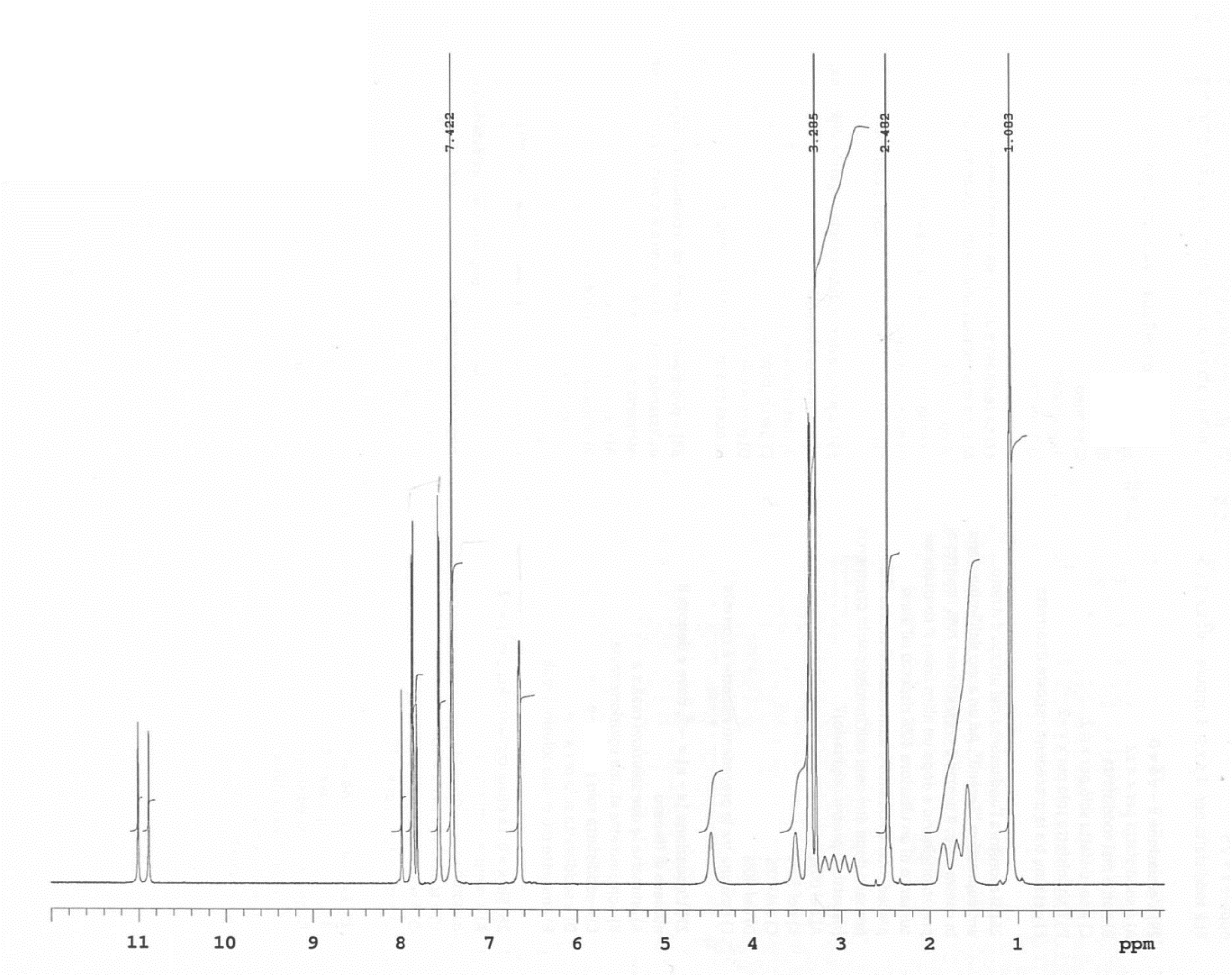

Supplement: Supplementary file 1 [file molecules-28-00091-s001.zip › molecules-2111073-supplementary.pdf]
